# A whole genome association study of mother-to-child transmission of HIV in Malawi

**DOI:** 10.1186/gm138

**Published:** 2010-03-01

**Authors:** Bonnie R Joubert, Ethan M Lange, Nora Franceschini, Victor Mwapasa, Kari E North, Steven R Meshnick, the NIAID Center for HIV/AIDS Vaccine Immunology

**Affiliations:** 1 Department of Epidemiology, Gillings School of Global Public Health, University of North Carolina, Chapel Hill, NC 27599, USA; 2 Department of Genetics, School of Medicine, University of North Carolina, Chapel Hill, NC 27599, USA; 3 Department of Biostatistics, Gillings School of Global Public Health, University of North Carolina, Chapel Hill, NC 27599, USA; 4 Carolina Center for Genome Sciences, University of North Carolina, Chapel Hill, NC 27599, USA; 5 College of Medicine, University of Malawi, Blantyre, Malawi

## Abstract

**Background:**

More than 300,000 children are newly infected with HIV each year, predominantly through mother-to-child transmission (HIV MTCT). Identification of host genetic traits associated with transmission may more clearly explain the mechanisms of HIV MTCT and further the development of a vaccine to protect infants from infection. Associations between transmission and a selection of genes or single nucleotide polymorphisms (SNP)s may give an incomplete picture of HIV MTCT etiology. Thus, this study employed a genome-wide association approach to identify novel variants associated with HIV MTCT.

**Methods:**

We conducted a nested case-control study of HIV MTCT using infants of HIV(+) mothers, drawn from a cohort study of malaria and HIV in pregnancy in Blantyre, Malawi. Whole genome scans (650,000 SNPs genotyped using Illumina genotyping assays) were obtained for each infant. Logistic regression was used to evaluate the association between each SNP and HIV MTCT.

**Results:**

Genotype results were available for 100 HIV(+) infants (at birth, 6, or 12 weeks) and 126 HIV(-) infants (at birth, 6, and 12 weeks). We identified 9 SNPs within 6 genes with a *P*-value < 5 × 10^-5 ^associated with the risk of transmission, in either unadjusted or adjusted by maternal HIV viral load analyses. Carriers of the rs8069770 variant allele were associated with a lower risk of HIV MTCT (odds ratio = 0.27, 95% confidence interval = 0.14, 0.51), where rs8069770 is located within *HS3ST3A1*, a gene involved in heparan sulfate biosynthesis. Interesting associations for SNPs located within or near genes involved in pregnancy and development, innate immunological response, or HIV protein interactions were also observed.

**Conclusions:**

This study used a genome-wide approach to identify novel variants associated with the risk of HIV MTCT in order to gain new insights into HIV MTCT etiology. Replication of this work using a larger sample size will help us to differentiate true positive findings.

## Background

In sub-Saharan Africa, over 1,300,000 pregnant women were living with HIV in 2007, 73,000 of which were in the small southern country, Malawi, landlocked between Tanzania, Zambia, and Mozambique, just North of Zimbabwe [1]. More than 300,000 children were newly infected with HIV in 2007, predominantly through mother-to-child transmission (HIV MTCT) [2]. Much of the risk of HIV MTCT can be reduced by treatment with single dose nevirapine (NVP). However, in many areas, mothers and their infants do not receive such regimens, and even in the context of prophylactic treatment, some infants become infected whereas others remain free of infection. Furthermore, HIV transmission can occur during pregnancy, labor and delivery, or through breastfeeding, by mechanisms which remain to be elucidated.

There is evidence for genetic variability in the mother and/or infant to be associated with susceptibility to HIV MTCT. However, a larger wealth of research describes genetic associations with adult HIV transmission and progression to AIDS. The following paragraphs note pertinent findings for various modes of HIV transmission and disease progression.

Alteration of viral entry has been implicated for several genes. One mechanism of cell entry involves HIV-1 binding with the CD4 receptor and co-receptor chemokine (CC motif) receptor 5 (CCR5). The CCR5 co-receptor also binds with chemokines produced by CD8+ T cells, including RANTES (CCL5), and MIP (macrophage inflammatory protein) 1α (CCL3) and 1β (CCL4). Higher concentrations of these ligands have been associated with a lower risk of HIV-1 infection and progression to AIDS, likely through competition with R5 strains of HIV for binding with the CCR5 receptor, preventing HIV from entering the cell and replicating [3-8]. Genes that regulate ligands for chemokine receptor genes have been associated with the risk of HIV infection, a notable example existing for chemokine (C-C motif) ligand 3-like 1 (*CCL3L1*). *CCL3L1 *copy number lower than population average has been associated with an increased risk of HIV transmission through different modes of transmission (adult and perinatal) and across various ethnic groups [9-13]. *CCL3L1 *copy number variation has also been associated with HIV/AIDS progression in adults [10,14-16].

Genes regulating co-receptor availability are also involved in HIV susceptibility. A prominent example in adults is the 32-base-pair deletion in the open reading frame of the *CCR5 *gene (*CCR5*-Δ32), where individuals homozygous for the Δ32 mutation are nearly resistant to infection by R5 strains [5-7,17,18]. However, the mutation does not always significantly alter susceptibility to maternal infection among infants [19]. The rarity of the Δ32 mutation in African populations [20], where HIV MTCT is more common, may account for this lack of association. It is possible that other *CCR5 *variations, such as the promoter polymorphisms 2459 (59029 or rs1799987) and 2135 (59353 or rs1799988), play stronger roles for HIV MTCT, when taking maternal HIV viral load into account [21]. *CCR5*-2132 (59356) has been noted for an increased risk of death among HIV-infected women, although the same study did not observe associations between *CCR5 *polymorphisms 2135 (59353), 2086 (59402 or rs1800023), and 2459 (59029 or rs1799987) and HIV MTCT [22].

Depending on the viral strain [23], HIV can use the CXC chemokine receptor 4 (CXCR4) as a co-receptor for CD4 for cell entry. Like CCR5, CXCR4 can be blocked by endogenous ligands [24,25]. The natural ligand for CXCR4 is the stromal cell-derived factor 1 (SDF1) [26-28], encoded by *SDF1 *(*CXCL12*). *SDF1-3-prime-A *has been associated with a reduced risk of HIV-1 infection [24,25], but not necessarily progression to AIDS [29,30] or HIV MTCT in African or other ancestry groups [31,32].

Intermediary receptors on dendritic or endothelial cells can be used by HIV-1 [33,34], and altered susceptibility to infection may result from polymorphisms in the genes regulating such receptors. This includes Dendritic cell-specific ICAM-grabbing non-integrin (*DC-SIGN*) [35-38] and syndecan genes such as *SDC-2 *[39]. High levels of *DC-SIGN *mRNA in the human placenta suggests a role for *DC-SIGN *for *in utero *transmission of HIV, even in the context of low maternal viral load [34]. Syndecans may be less important alone as they are when connected with other factors such as chemokine receptors or heparan sulfate. For example, the SDC-4/CXCR4 complex binds with SDF-1 [40], which can alter HIV binding. The syndecan protein bound with heparan sulfate (proteoglycan) can also bind with gp120 of HIV-1 [41], which may facilitate HIV-1 cell entry [42] or cell-free transport [43]. There are multiple genes encoding syndecans and heparan sulfate proteglycans that remain to be clearly described in relation to HIV MTCT.

Finally, genes involved in the host immune response can play a role in HIV/AIDS susceptibility. The valine to isoleucine substitution at codon 64 in the chemokine co-receptor 2b gene (*CCR2-V64I*) demonstrates linkage disequilibrium with the *CCR5 *promoter region [44] and is common in populations of African ancestry [44-46]. The natural ligand of *CCR2 *is CCL2 (MCP-1), which does not bind with CCR5 or CXCR4 [47]. *CCR2-V64I *is associated with delayed disease progression in adults, but with variable replication [44,48-50]. It is possible that the *CCR2 *gene does not individually influence HIV progression to AIDS, but rather, acts in combination with other gene polymorphisms such as the variants of *CCR5*, *CXCR4*, and possibly human leukocyte antigen (*HLA*) gene variants [51] in promoting or preventing infection. It has been suggested that activation of the immune system rather than receptor blockage explains the association with HIV/AIDS [47].

A variety of *HLA *gene variants are associated with susceptibility to HIV/AIDS in adults. This includes *HLA *complex P5 (*HCP5*) rs2395029 (in strong linkage disequilibrium with HLA-B*5701) and *HLA-C *rs926942 associated with HIV viral set point [52] in a genome-wide association study, *HLA-Bw4 *associated with a lower risk of heterosexual HIV transmission [53], and HLA-B*35 alone [54,55] or in combination with HLA-Cw*04 [56] associated with disease progression. An epistatic interaction between *HLA-B Bw4-80I *and activating killer immunoglobulin-like receptors (*KIR*) variant *KIR3DS1 *has also been associated with a protection from rapid progression to AIDS [57,58], likely through increases in natural killer cell activity, cell lysis, and subsequent reduction in viral load [57].

More pertinent to HIV MTCT are *HLA *variants evaluated in pregnant women or maternal-fetal polymorphism mismatches in *HLA *variants, which can protect infants from infection. One study found that mothers with *HLA-B *variants (*1302, *3501, *3503, *4402, *5001) transmitted HIV to their infant even in the context of low viral loads, whereas mothers with other variants (*4901, *5301) did not transmit the virus despite high viral loads [59]. Furthermore, mother-infant pairs discordant with regards to the *HLA-G *variants *3743C/T*, *634C/G*, or *714insG/G *have been shown to experience a lower risk of HIV MTCT compared to concordant mother-child pairs [60].

The *MBL2 *gene plays a role in the innate immune responses to infection and encodes the mannose-binding lectin (MBL) protein [61-64]. Several *MBL2 *polymorphisms can result in MBL deficiency, which has been associated with increased risk of HIV MTCT [65]. Apolipoprotein B mRNA Editing Catalytic Polypeptide 3 g (*APOBEC3G*), inhibits HIV-1 replication [66] and is associated with disease progression in children [67]. However, the association between *APOBEC3G *variants in the risk of HIV MTCT has not been established.

It is possible that the genetic risk factors involved in HIV infection and disease progression in adults do not directly overlap with the HIV MTCT phenotype and that the mechanisms with genetic underpinnings for HIV MTCT await discovery. It is also likely that what we know about HIV MTCT genetic risk factors is only one piece of the puzzle. To uncover new genes associated with HIV MTCT, we conducted a whole genome scan for fetal susceptibility to maternal HIV infection using information from consenting mother-infant pairs receiving antenatal care in Blantyre, Malawi, a population with a high burden of HIV/AIDS.

Because HIV MTCT is a rare phenotype, it is difficult to ascertain thousands of cases in order to obtain adequate power for a typical genome-wide association study. However, genome-wide approaches for such a phenotype can still be fruitful for furthering our understanding of HIV MTCT etiology and for generating hypotheses. Where possible, we also report the effects of SNPs within genes known to be associated with HIV/AIDS, for the purposes of replication in our study population.

## Methods

### Study design and population

The study participants were a subset of a larger prospective cohort study of malaria and HIV in pregnancy [68,69]. The cohort was conducted from 2000 to 2004 and included 3,825 consenting pregnant women admitted to Queen Elizabeth Central Hospital in Blantyre, Malawi, as previously described [69]. HIV-infected women and their infants received a single dose (200 mg) of NVP at the onset of labor or at the time of delivery, respectively. A total of 1,157 women tested positive for HIV, 884 of which delivered at Queen Elizabeth Central Hospital, resulting in 807 singleton live births. At delivery, 751 infants were tested for HIV, identifying 65 HIV positive infants at birth. Of the 686 HIV negative infants, 179 were lost to follow-up. The remaining 507 HIV negative infants were tested for HIV at 6 and 12 weeks, resulting in 89 additional HIV positive infants. Based on mother reports, 98.4% and 96.5% of infants were breast fed at 6 and 12 weeks postpartum, respectively.

In order to evaluate infant susceptibility to maternal HIV infection, a nested case control was conducted, focusing on infants of HIV positive mothers. Given that all such infants were HIV-exposed, cases were defined as infants who became HIV positive at birth, 6 weeks, or 12 weeks. Controls were defined as infants who remained HIV negative at all visits. Genotyping was performed for as many cases as possible. We first evaluated samples for sufficient DNA for genome-wide genotyping, which was obtainable for 115 of the 154 cases. Funding and supplies were only available to test an approximately 1:1 case:control ratio. We selected controls in a slightly higher than 1:1 case:control ratio, anticipating loss of samples due to insufficient DNA. A total of 203 of the 418 controls were selected using simple random selection in *STATA *version 10 [70], 153 of which had sufficient DNA. The controls had a similar distribution across time of enrollment as the cases. The total sample size subjected to genotyping was 268 infants (115 cases + 153 controls) of HIV positive mothers. Because the control status of subjects was designated at the beginning of sample selection for the nested case control, this study was analyzed as a case-cohort study [71]. Mothers of infants could not be genotyped as the original institutional review board approval did not include this. It was not possible to return to study participants in order to obtain informed consent for maternal genotyping. Thus, no test of transmission disequilibrium or analyses involving mother-infant pairs could be conducted. The focus was infant genomic susceptibility to HIV infection, given an HIV positive mother. The original cohort study obtained consent from study participants to collect and use samples for biological measurements including but not limited to diagnosis of disease and for genotyping. Written informed consent forms were available in both English and Chichewa, the predominant language in Malawi. This study was approved by the Malawi College of Medicine Research and Ethics Committee and by the institutional review board at the University of North Carolina at Chapel Hill. Modification of the original institutional review board approval was obtained to ensure the approval of large-scale genotyping of SNPs across the genome.

### Power analysis

Power was calculated based on a genome-wide scan of approximately 587,000 SNPs, as over 68,000 SNPs were removed due to quality control. Per specifications of the software Quanto [72], power was computed using a log-additive model, varying allele frequency (10 to 30%), a baseline risk of 25% (to approximate the proportion of infants that became infected with HIV from HIV positive mothers in the genome wide association study population), a case to control ratio of 1:1, and an Bonferroni adjusted *P*-value of 0.05/600,000 SNPs = 1 × 10^-8 ^to account for multiple testing. Power was estimated for varying relative risks (1.25 to 3.25).

### Genotyping

Infant genotyping was performed at Duke University Genotyping Core Laboratories, by using Illumina's HumanHap650Y Genotyping BeadChip. This BeadChip enables whole-genome genotyping of over 655,000 tagSNPs derived from the International HapMap Project [73] and over 100,000 tag SNPs selected based on the Yoruban Nigerian HapMap population. The BeadChip contains over 4,300 SNPs with copy number polymorphism regions of the genome, 8,000 non-synonymous SNPs, 1,800 tag SNPs in the major histocompatibility complex important for immunological relevance, 177 mitochondrial SNPs, and 11 Y-chromosome SNPs.

### Quality control

The quality control for genotyping error was performed at Duke University Genomic Laboratories as previously described [52]. Briefly, all samples were brought into a BeadStudio data file and clustering of samples was evaluated in order to determine random clustering of SNPs. Samples with very low call rates (<95%) or insufficient DNA concentration were excluded. Subsequent reclustering of undeleted SNPs and additional exclusion by call rate was performed [52]. SNPs with a Het Excess value between -1.0 to -0.1 and 0.1 to 1.0 were evaluated to determine if raw and normalized data indicated clean calls for the genotypes [52].

Statistical quality control was performed at the University of North Carolina at Chapel Hill. Individuals missing more than 10% of marker data, SNPs missing more than 10% of genotypes, SNPs with a minor allele frequency (MAF) ≤ 0.01, and SNPs out of Hardy-Weinberg equilibrium (HWE) (*P *< 0.001) in the control group were excluded. Gender verification was completed for all subjects to ensure that gender recorded in the covariate dataset matched with gender based on genetic data. For mismatched or missing gender, gender was imputed based on the X chromosome (N = 9). Related individuals were identified by first estimating identity by descent (IBD). A minimal list of individuals with estimated genome-wide IBD proportions > 0.05 with at least one included subject were removed (N = 5). Statistical quality control was performed in PLINK version 1.05 [74]. Analyses were run without exclusions due to HWE in order to assess the difference in results.

### Statistical analysis

Assuming an additive genetic model, logistic regression was performed where the outcome of interest was HIV status of the infant (positive or negative). The null hypothesis was that the SNP of interest was not associated with HIV MTCT: Ho: β1 = 0, compared to the alternative hypothesis, that the SNP was associated with HIV MTCT: Ha: β1 ≠ 0. All SNPs were assumed to be independent, and Bonferroni correction was used to adjust for multiple testing. Odds ratios (ORs) were obtained to approximate the risk ratios. These statistical analyses were conducted in PLINK version 1.05 [74] and the results were visualized in WGAViewer version 1.26F [75].

Logistic regression was adjusted for maternal viral load (MVL), as it is a key risk factor for HIV MTCT. MVL could not be evaluated for effect measure modification because of the small sample size. Logistic regression results were presented for both unadjusted and MVL adjusted analyses. We also investigated maternal syphilis for significant confounding, although the number of infants of HIV positive mothers who also had syphilis was small (N = 20). We did not evaluate maternal malaria for confounding as it was not associated with the outcome, HIV MTCT [68,69]. In order to evaluate population stratification, principal components analysis was performed by using EIGENSOFT version 2.0 [76,77]. Principal component(s) (PCs) were then evaluated for association for SNPs associated with HIV MTCT. PCs were determined to represent potential confounders if they were associated with both the SNP of interest and HIV MTCT. If necessary, logistic regression was repeated adjusting for confounding PCs.

In order to evaluate the consistency of associations by mode of transmission, we evaluated each SNP for association with intrauterine and intrapartum transmission. Intrauterine transmission was estimated by infant HIV status at birth. Intrapartum transmission was assigned to infants who were HIV negative at birth but who became HIV positive at week 6. Transmission through breastfeeding was estimated at week 12. For each mode of transmission, the results for SNPs within key genes previously associated with HIV/AIDS were summarized.

## Results

### Quality control and power analysis

A total of 246 infants (114 cases, 132 controls; 116 males, 121 females, 9 with imputed gender) passed laboratory quality control. Statistical quality control removed 15 individuals for low genotyping and 5 who had estimated genome-wide IBD proportions > 0.05 with at least one included subject. This resulted in a total of 226 individuals (100 cases, 126 controls; 112 males, 114 females). Of the 655,352 SNPs tested, 68,671 failed statistical quality control due to HWE *P *< 0.001 in the controls (N = 425), low genotyping rate (N = 21,589), or for MAF <0.01 (N = 53,477), where some overlap of SNPs across exclusion criteria existed. Results are summarized for 586,681 SNPs.

No evidence of population stratification was present (Eigen value range: 0.817 to 1.20, mean = 0.995, genomic inflation factor based on median χ^2 ^= 1.023, mean χ^2 ^= 1.013). The power analyses estimated that with a *P*-value of 1 × 10^-8^, a baseline risk of 25%, and an allele frequency of 10%, our power was ≤32% and 58% for a relative risk (RR) of ≤3.0 and 3.5, respectively. For an allele frequency of 20%, this changed to 10%, 50%, 85%, and 97%, for RR = 2.0, 2.5, 3.0, and 3.5, respectively. And for an allele frequency of 30%, this changed to 22%, 75%, 96%, and 99%, for RR = 2.0, 2.5, 3.0, and 3.5, respectively. This implies that our genome-wide association dataset with a sample size of 226 is powered to detect large effects of very common variants, but underpowered to detect small effects of rare variants. Because additional cases could not be obtained, we were unable to increase sample size in order to boost power. Rather, limited genome-wide statistical significance was noted.

### Association results

**Figure 1:**
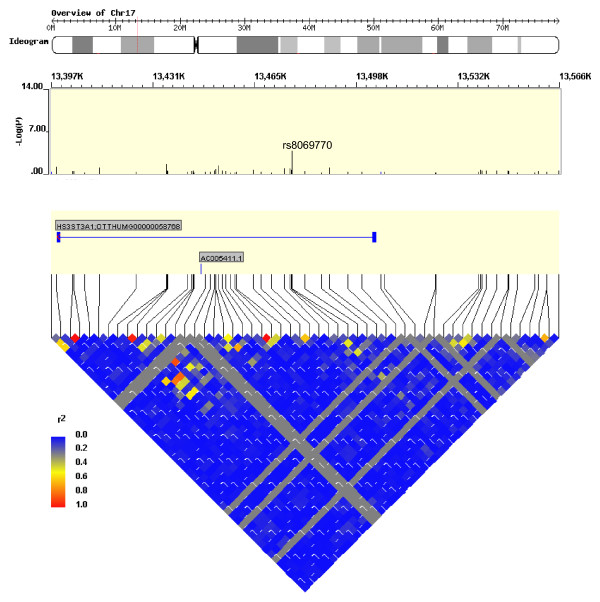
**Map of the *HS3ST3A1 *gene on chromosome 17**. Position and -log(*p*) of SNPs in the region are displayed, including the SNP rs8069770 with the highest -log(*p*). Triangle display of linkage disequilibrium across SNPs corresponds to r^2 ^estimates. Plot constructed using WGAViewer software version 1.26F.

Although no genome-wide significant SNPs were detected (*P *< 1 × 10^-7^), we identified nine SNPs within six genes with a *P*-value < 5 × 10^-5 ^in either unadjusted analyses and/or analyses adjusted by MVL (Table 1). Adjustment by maternal syphilis made little impact on the effect estimates or statistical significance (data not shown). Several of the 50 most significant SNPs were located within interesting genes, including 7 SNPs near or within genes involved in pregnancy and development (Table 2). An additional 7 SNPs were located near or within genes with immunological function and/or HIV-1 protein interactions (Table 3). One of the top SNPs corresponding to functional interest was rs8069770, located within the gene heparan sulfate (glucosamine) 3-*O*-sulfotransferase 3A1 (*HS3ST3A1*; Figure 1). Analyses run including SNPs out of HWE in the control group gave similar results (data not shown). None of the ten PCs evaluated were associated with rs8069770 (*P *= 0.763, 0.977, 0.715, 0.447, 0.320, 0.714, 0.523, 0.958, 0.696, 0.902). Thus, subsequent adjustment by PCs was not necessary.

**Table 1:** HIV MTCT association results for SNPs, selected by *P*-value

CHR	SNP^type^	A1	A2	MAF	Unadjusted OR (95% CI)	*P*	Adjusted OR (95% CI)	*P*	Nearest gene
17	rs12306^a^	A	G	0.23	0.33 (0.20, 0.55)	2.02E-05	0.34 (0.20, 0.57)	3.92E-05	WD repeat and SOCS box-containing 1 (*WSB1*)
8	rs476321^a^	T	C	0.27	2.55 (1.65, 3.92)	2.15E-05	2.50 (1.62, 3.87)	3.42E-05	Protein coding, protein info: transcription factor CP2-like 3, deafness, autosomal dominant 28, grainyhead-like 2 (Drosophila) (*GRHL2*)
6	rs2268993^a^	C	G	0.27	2.71 (1.71, 4.28)	2.20E-05	2.70 (1.70, 4.28)	2.65E-05	Solute carrier family 35 (CMP-sialic acid transporter), member A1 (*SLC35A1*)
18	rs8084223^b^	T	C	0.15	0.26 (0.14, 0.49)	3.41E-05	0.26 (0.14, 0.50)	4.21E-05	*AC104961.7*
23	rs5934013^a^	G	A	0.15	4.18 (2.12, 8.24)	3.61E-05	4.09 (2.08, 8.06)	4.68E-05	FERM and PDZ domain containing 4 (*FRMPD4*)
8	rs9314565^b^	G	C	0.47	0.42 (0.27, 0.63)	4.13E-05	0.41 (0.27, 0.63)	3.64E-05	*AC019176.4*
3	rs4234621^b^	C	T	0.28	0.39 (0.25, 0.61)	5.03E-05	0.38 (0.24, 0.60)	4.58E-05	Pyrin domain containing 2 (*PYDC2*)
14	rs2287652^a^	C	A	0.2	0.32 (0.19, 0.56)	5.15E-05	0.33 (0.19, 0.57)	7.12E-05	aarF domain containing kinase 1 (*ADCK1*)
9	rs1889055^b^	C	N/A	0.24	2.52 (1.61, 3.93)	5.21E-05	2.48 (1.59, 3.87)	6.32E-05	*RP11-48L13.1*
7	rs216743^a^	A	G	0.1	4.22 (2.09, 8.53)	6.16E-05	4.23 (2.08, 8.61)	6.89E-05	cAMP responsive element binding protein 5 (*CREB5*)
7	rs216744^a^	G	G	0.1	4.22 (2.09, 8.53)	6.16E-05	4.23 (2.08, 8.61)	6.89E-05	cAMP responsive element binding protein 5 (*CREB5*)
22	rs131817^a^	T	G	0.23	0.37 (0.22, 0.60)	6.68E-05	0.36 (0.22, 0.59)	6.62E-05	Non-SMC condensin II complex, subunit H2 (*NCAPH2*)
7	rs4722999^a^	C	C	0.32	2.46 (1.58, 3.84)	7.07E-05	2.38 (1.52, 3.72)	1.49E-04	Corticotropin releasing hormone receptor 2 (*CRHR2*)
17	rs8069770^a^	T	G	0.14	0.27 (0.14, 0.51)	7.17E-05	0.25 (0.13, 0.49)	3.79E-05	Heparan sulfate (glucosamine) 3-O-sulfotransferase 3A1 (*HS3ST3A1*)
5	rs6884962^c^	G	A	0.49	2.18 (1.48, 3.21)	7.31E-05	2.15 (1.46, 3.17)	1.07E-04	*AC008412.8*
12	rs12579934^a^	T	A	0.46	2.24 (1.49, 3.36)	9.59E-05	2.48 (1.63, 3.78)	2.45E-05	Branched chain aminotransferase 1, cytosolic (*BCAT1*)
9	rs12376718^b^	T	A	0.15	3.07 (1.75, 5.39)	9.79E-05	2.97 (1.69, 5.20)	1.47E-04	*RP11-48L13.1*
16	rs6540013^b^	G	C	0.39	0.45 (0.30, 0.68)	1.16E-04	0.44 (0.29, 0.66)	8.99E-05	*AC010531.8*
16	rs12598821^a^	T	T	0.48	0.45 (0.30, 0.68)	1.20E-04	0.43 (0.28, 0.65)	6.65E-05	*AC010333.7*
1	rs3861824^b^	A	G	0.11	0.23 (0.11, 0.50)	1.98E-04	0.20 (0.09, 0.44)	6.29E-05	Disabled homolog 1 (Drosophila) (*DAB1*)

Top 20 most significant SNPs based on *P*-values from crude and/or adjusted by maternal HIV viral load analyses, sorted by unadjusted *P*-value. CHR, chromosome; SNP^type^, SNP and type, where type refers to the position of the SNP relative to the closest gene (^a^intronic, ^b^intergenic, ^c^upstream); A1, risk allele designated by PLINK; A2, major allele; MAF, minor allele frequency; OR, odds ratio; 95% CI, 95% confidence interval of the OR; Adjusted OR, OR from analyses adjusted by maternal HIV viral load.

**Table 2:** Top SNPs in or near genes with roles in pregnancy and development

CHR	SNP^type^	*P*	Nearest gene	Presumed gene function
17	rs8069770^a^	3.79E-05	Heparan sulfate (glucosamine) 3-O-sulfotransferase 3A1 (*HS3ST3A1*)	Abundant expression in placenta; HIV-1 requires the gene product heparan sulfate proteoglycans for uptake in trophoblasts (cells forming the placental barrier); involved in biosynthesis of an entry receptor for herpes simplex virus 1
17	rs12306^a^	3.92E-05	WD repeat and SOCS box-containing 1 (*WSB1*)	Unknown protein function induced by Hedgehog signaling in embryonic structures during chicken development
4	rs1433666^a^	1.00E-04	Glutamate receptor, ionotropic, delta 2 (*GRID2*)	Homozygosity for this mutation in mice results in death shortly after birth, related to the loss of mid- and hindbrain neurons during late embryogenesis
5	rs6884962^b^	1.00E-04	NK2 transcription factor related, locus 5 (Drosophila) (*NKX2-5*)	Regulates tissue-specific gene expression essential for tissue differentiation; regulates temporal and spatial patterns of development
7	rs4722999^a^	1.00E-04	Corticotropin releasing hormone receptor 2 (*CRHR2*)	Detected in placenta, myometrium, decidua, and fetal membranes; expression is down-regulated in uterine tissues during pregnancy, most pronounced in laboring cervix; suggested paracrine role in birth process (for example, effects on macrophages and endothelial cells)
2	rs2677510^b^	3.00E-04	GLI-Kruppel family member GLI2 (*GLI2*)	Role during embryogenesis, DNA binding, and Sonic hedgehog (Shh) signaling to oncogenes in embryonal carcinoma cells
6	rs2268447^a^	4.00E-04	Pleiomorphic adenoma gene-like 1 (*PLAGL1*)	Mutations associated with congenital abnormalities, potential role in ovarian and other types of cancer; genetically imprinted in neonatal diabetes

The sources of the presumed gene function are NCBI Entrez Gene and OMIM [88,94]. CHR, chromosome; SNP^type^, SNP and type, where type refers to the position of the SNP relative to the closest gene (^a^intronic, ^b^intergenic); *P*, adjusted by maternal HIV viral load *P*-value.

**Table 3:** Top SNPs in or near genes with immunological function or HIV-1 protein interactions

CHR	SNP^type^	*P*	Nearest gene	Presumed gene function‡
6	rs3131036^a^	2.00E-04	Discoidin domain receptor family, member 1 (*DDR1*)	Proximal to *HLA *genes; interacts w/collagen to up-regulate *IL-8 *and inflammatory macrophages.
10	rs3124199^b^	2.00E-04	Mitogen-activated protein kinase kinase kinase 8 (*MAP3K8*)	Promotes production of TNF-alpha and IL-2 during T lymphocyte activation; promotes proteolysis of cytokine activator NFKB1 in rats.
4	rs1358594^b^	3.00E-04	Interleukin 8 (*IL8*)	HIV-1 Nef, Tat, and Vpr regulate *IL-8 *expression. IL-8 protein mediates inflammatory response including CD4+ response to HIV-1 infection.
7	rs10254544^c^	3.00E-04	Nucleoporin like 2 (*NUPL2*)	Interacts with HIV-1 Rev to mediate nuclear import of viral DNA and inhibit nuclear export of HIV-1 mRNA.
9	rs302923^d^	3.00E-04	General transcription factor IIIC, polypeptide 4, 90kDa (*GTF3C4*)	HIV-1 Tat upregulates RNA polymerase III transcription by activating this gene.
20	rs6037908^b^	3.00E-04	Prion protein (p27-30) (Creutzfeldt-Jakob disease, Gerstmann-Strausler-Scheinker syndrome, fatal familial insomnia) (*PRNP*)	HIV-1 Tat binds to a stem-loop structure in the mRNA of PrP, upregulating expression.
7	rs6951646^b^	4.00E-04	Sp4 transcription factor (*SP4*)	Activates the HIV-1 LTR promoter, possibly enhancing HIV-1 Tat-mediated transactivation of the viral promoter.

The sources of the presumed gene function are NCBI Entrez Gene and OMIM [88,94]. CHR, chromosome; SNP^type^, SNP and type, where type refers to the position of the SNP relative to the closest gene (^a^intronic, ^b^intergenic, ^c^upstream, ^d^downstream); *P*, adjusted by maternal HIV viral load *P*-value.

For the top 20 most significant SNPs summarized in Table 1, we evaluated the effect estimates and statistical significance for intrauterine and intrapartum HIV transmission (Additional file 1). We were unable to include results for transmission through breastfeeding because the outcome was too rare. For all SNPs described, the direction of effect (higher risk or lower risk of HIV transmission) was consistent across mode of transmission (Additional file 1). The results for SNPs within 10 kb of key genes of interest were also reported (Additional file 2). We were unable to report results specific to the marker for the *CCL3L1 *copy number variation, rs72248989, but we report the effects of SNPs in this region (Additional file 2).

## Discussion

We conducted a genome-wide association study to identify genetic variants that may influence HIV MTCT. Although limited by sample size and the power to detect genome-wide statistical significance, we were powered to detect large genetic effects for common variants (effect estimate >3.0, MAF >20% or effect estimate >2.5, allele frequency >25%). No such genome-wide statistically significant genetic effects were detected. Nonetheless, several findings were notable and may offer supportive data for other studies of the genetics of HIV MTCT.

Several SNPs with biological significance were noted. One of these is the SNP rs8069770, located within the gene *HS3ST3A1*. This gene encodes the enzyme 3-*O*-sulfotransferase, which catalyzes the biosynthesis of a specific subtype of heparan sulfate (HS), 3-*O*-sulfated heparan sulfate. This HS subtype has specific functional significance for herpes simplex virus-1 [78,79]. Although HS has been shown to be involved in HIV infection [80-83], to our knowledge, no sub-type-specific investigations of HS have been conducted for association with HIV MTCT. Furthermore, HIV-1 virus [41,84] and the chemokine RANTES [41,85,86] have been noted to bind to syndecans, which are core transmembrane proteins capable of carrying HS [87]. It is possible that specific or multiple components of HS proteoglycans, which consist of the bound core protein attached to HS, are involved in HIV MTCT. We suggest two possible mechanisms: the attachment of HS proteoglycans to HIV could prevent the virus from crossing the placenta and possibly facilitate viral sequestration in the placenta; or, HS proteoglycans binding with RANTES could leave *CCR5 *receptors available to bind with HIV virus and facilitate transmission across the placenta. The former mechanism would agree with the direction of effect we observed for rs8069770. However, much more research is needed in order to more clearly develop mechanistic hypotheses involving HS, at both the genetic level regulating the biosynthesis of HS subtypes, and at the protein level. We observed that the frequency of the minor allele of rs8069770 among cases/controls was similar across transmission type: case/control frequencies were 0.07/0.19, 0.07/0.16, and 0.09/0.18 for cumulative HIV MTCT, intrauterine transmission, and intrapartum transmission, respectively. The direction of effect was also consistent across transmission category (Additional file 1), suggesting that the mechanism may not be specifically localized to the placenta.

Two SNPs were located within genes involved in embryonic development in animal models [88]: rs12306 (*P *= 3.29 × 10^-5^) within the WD repeats and SOCS box-containing 1 (*WSB1*) gene, and rs1433666 (*P *= 0.0001) within the Glutamate receptor, ionotropic, delta 2 (*GRID2*) gene. The role of *WSB1 *in human embryonic development or in the risk of HIV MTCT is not well described. *GRID2 *has been noted as a large region of genomic instability (fragile site) and has been associated with cancer and neural development [89,90]. Subsequent studies of these genes in humans would be valuable, in particular for probing roles in viral infection.

There were two SNPs (rs216743 and rs216744) with *P*-values < 7 × 10^-5 ^identified in the cAMP response element binding protein 5 gene (*CREB5*). The *CREB5 *product is part of the CRE (cAMP response element)-binding protein family. One member of this family, CRE-BP1, is involved in mediating the adenovirus E1A-induced trans-activation [91]. *CREB5 *has also been noted to serve as an integration site for xenotropic murine leukemia virus-related virus (XMRV) in prostate cancer tissue from patients homozygous for a reduced activity variant of the antiviral enzyme RNase L [92]. Another SNP, rs1358594 (*P *= 0.0003), was of interest as it is within *IL8*, which mediates inflammatory response to HIV-1 infection [88]. Six other SNPs were found within genes that play a role in HIV infection. This may be suggestive of similar roles for such genes in HIV MTCT.

The Illumina 650Y BeadChip methodology provides genotypes of predominantly biallelic SNPs that are approximately evenly spaced across the genome rather than selected based on known functional significance. This limited our ability to replicate associations between some regions of interest (that is, *CCR5*) and HIV MTCT in this study. We were also unable to directly evaluate some key copy number variations (that is, *CCL3L1*) for association with HIV MTCT. However, we do describe the results for SNPs within 10 kb of the key genes associated with HIV/AIDS, including the association between SNPs close to the marker for the *CCL3L1 *copy number variation rs71148989 (Additional file 2). Our small sample size may also have limited our ability to detect statistically significant associations in some regions of interest, in particular for small effects.

We did not describe the most statistically significant SNPs (potentially different sets of top SNPs) by mode of transmission because of the small number of cases by transmission type. Rather, we compare the results for top SNPs from cumulative HIV MTCT analyses across other modes of transmission (intrauterine/intrapartum; Additional file 1) to assess consistency. Because the number of transmission events through breastfeeding was very rare (N = 10), we were unable to report the associations specific to postpartum transmission. We observed consistent direction of effects (higher/lower risk of HIV MTCT) across mode of transmission, which suggests that the effects of the top SNPs are not specific to biological events taking place *in utero*. However, for some SNPs, the strength of effect differed across transmission type. For example, rs5934013 of FERM and PDZ domain containing 4 (*FRMPD4*) was associated with a higher risk of HIV MTCT (MVL-adjusted OR = 4.09, 95% confidence interval (CI) = 2.08, 8.06), also found for intrauterine transmission (MVL-adjusted OR = 1.83, 95% CI = 0.96, 3.47), and intrapartum transmission (MVL-adjusted OR = 3.39, 95% CI = 1.46, 7.85). The stronger effect size for intrapartum compared to intrauterine transmission is interesting, possibly useful for developing mechanistic hypotheses, but warrants caution with interpretation due to sample size.

We previously noted that all mothers in the study received NVP, in accordance with the HIVNET 012 protocol [93]. This may limit the generalizability of our findings to populations with different drug treatment or with no drug treatment during pregnancy or after delivery. It may also have limited our ability to replicate or identify novel SNP associations with HIV MTCT that are only present in the absence of treatment. However, because NVP treatment was administered to all subjects, this study may more clearly illustrate the genetic effects that are strong enough to maintain association with HIV MTCT even in the context of NVP. Such effects may be of greater interest for therapeutic applications or for pharmacogenomic research efforts.

Due to the nature and frequency of this rare HIV MTCT phenotype, we were unable to ascertain a sufficient number of cases to be powered to establish genome-wide statistical significance. However, this study did provide some new insights into the genetics of HIV MTCT and aims to facilitate future genetic studies for this phenotype.

## Conclusions

This study evaluated over 586,000 SNPs for association with HIV MTCT in a set of HIV-exposed infants from Blantyre, Malawi. Although we were unable to detect genome-wide statistically significant effects, several SNPs with *P*-values < 5 × 10^-5 ^with biological significance were noted. Replication of this work using a larger sample size will help us to differentiate true positive findings.

## Abbreviations

CI: confidence interval; CRE-BP: cAMP response element-binding protein; HLA: human leukocyte antigen; HS: heparan sulfate; HWE: Hardy-Weinberg equilibrium; IBD: identity by descent; MAF: minor allele frequency; MTCT: mother-to-child transmission; MVL: maternal viral load; NVP: nevirapine; OR: odds ratio; RANTES: regulated upon activation, normal T cell expressed and secreted; PC: principal component; RR: relative risk; SNP: single nucleotide polymorphism.

## Competing interests

The authors declare that they have no competing interests.

## Authors' contributions

BRJ completed the statistical analysis, writing of the manuscript, and contributed to the intellectual content of the study. EL contributed to the statistical analysis and intellectual content. NF contributed to the intellectual content and revisions of the manuscript. VM was involved in the original cohort design and data collection. KEN was involved in the intellectual content, statistical analysis, and manuscript revisions. SRM was involved in the original cohort design and data collection, provided project mentorship, and contributed to the intellectual content and manuscript revisions.

## Supplementary Material

Additional file 1 A Word document giving effect estimates for top SNPs of interest, by mode of transmission. The data provided represent the genome-wide association analysis by mode of HIV transmission. Click here for file

Additional file 2 A Word document giving effect estimates for SNPs near or within genes associated with HIV/AIDS. The data provided represent the genome-wide association analysis for specific regions that have previously demonstrated association with HIV/AIDS, described in the Introduction section. Click here for file
