# Correction: A whole genome association study of mother-to-child transmission of HIV in Malawi

**DOI:** 10.1186/gm197

**Published:** 2010-10-11

**Authors:** Bonnie R Joubert, Ethan M Lange, Nora Franceschini, Victor Mwapasa, Kari E North, Steven R Meshnick

**Affiliations:** 1 Department of Epidemiology, Gillings School of Global Public Health, University of North Carolina, Chapel Hill, NC 27599, USA; 2 Department of Genetics, School of Medicine, University of North Carolina, Chapel Hill, NC 27599, USA; 3 Department of Biostatistics, Gillings School of Global Public Health, University of North Carolina, Chapel Hill, NC 27599, USA; 4 Carolina Center for Genome Sciences, University of North Carolina, Chapel Hill, NC 27599, USA; 5 College of Medicine, University of Malawi, Blantyre, Malawi

## Abstract

A correction to: Bonnie R Joubert, Ethan M Lange, Nora Franceschini, Victor Mwapasa, Kari E North, Steven R Meshnick andthe NIAID Center for HIV/AIDS Vaccine Immunology. **A whole genome association study of mother-to-child transmission of HIV in Malawi**. *Genome Medicine *2010, **2**:17.

## 

We wish to report some corrections to our study [1], none of which alters the interpretation of the data or the conclusions drawn. After publication of this work we noted that the sample size required further clarification.

## Results

A total of 246 infants (114 cases, 132 controls; 116 males, 121 females, 9 with imputed gender) passed laboratory quality control. Statistical quality control removed 15 individuals for low genotyping, resulting in a total of 231 individuals (103 cases, 128 controls; 114 males, 117 females). Of the 655,352 SNPs tested, 68,297 failed statistical quality control due to HWE P < 0.001 in the controls (N = 431), low genotyping rate (N = 21,589), or for MAF < 0.01 (N = 53,477), where some overlap of SNPs across exclusion criteria existed. Results were presented for 587,055 SNPs.

## Additional data files

Additional data files 1 and 2 containing the corrected data are available online with this article.

## Supplementary Material

Additional file 1 **A Word document giving effect estimates for top SNPs of interest, by mode of transmission**. The data provided represent the genome-wide association analysis by mode of HIV transmission. Click here for file

Additional file 2 **A Word document giving effect estimates for SNPs near or within genes associated with HIV/AIDS**. The data provided represent the genome-wide association analysis for specific regions that have previously demonstrated association with HIV/AIDS, described in the Introduction section. Click here for file
