# Supplementary material for: A whole genome association study of mother-to-child transmission of HIV in Malawi
Source: Genome Med. 2010 Mar 1;2(3):17. doi: 10.1186/gm138 (PMC2873795; doi:10.1186/gm138)
Supplement: Additional file 1 — A Word document giving effect estimates for top SNPs of interest, by mode of transmission. The data provided represent the genome-wide association analysis by mode of HIV transmission. [file gm138-S1.DOCX]

Additional file 1. Effect estimates for top SNPs of interest, by mode of transmission

|  |  |  | **Intrauterine Transmission** | | | **Intrapartum Transmission** | | |
| --- | --- | --- | --- | --- | --- | --- | --- | --- |
| **CHR** | **SNP** | **A1** | **MAF** | **OR (95%CI)** | ***p*** | **MAF** | **OR (95% CI)** | ***p*** |
| 17 | rs12306 | A | 0.23 | 0.70 (0.38, 1.29) | 2.56E-01 | 0.23 | 0.33 (0.15, 0.72) | 5.52E-03 |
| 8 | rs476321 | T | 0.27 | 1.48 (0.91, 2.40) | 1.12E-01 | 0.27 | 2.39 (1.33, 4.32) | 3.75E-03 |
| 6 | rs2268993 | C | 0.27 | 1.37 (0.80, 2.34) | 2.48E-01 | 0.27 | 3.41 (1.72, 6.74) | 4.26E-04 |
| 18 | rs8084223 | T | 0.15 | 0.30 (0.12, 0.75) | 9.77E-03 | 0.15 | 0.26 (0.10, 0.69) | 6.46E-03 |
| 23 | rs5934013 | G | 0.15 | 1.83 (0.96 , 3.47) | 6.53E-02 | 0.15 | 3.39 (1.46, 7.85) | 4.42E-03 |
| 8 | rs9314565 | G | 0.47 | 0.47 (0.28 , 0.79) | 4.61E-03 | 0.47 | 0.37 (0.20, 0.69) | 1.68E-03 |
| 3 | rs4234621 | C | 0.29 | 0.81 (0.47, 1.39) | 4.44E-01 | 0.29 | 0.17 (0.07, 0.42) | 1.14E-04 |
| 14 | rs2287652 | C | 0.20 | 0.55 (0.28, 1.09) | 8.74E-02 | 0.20 | 0.40 (0.18, 0.89) | 2.54E-02 |
| 9 | rs1889055 | C | 0.23 | 1.94 (1.18, 3.21) | 9.18E-03 | 0.23 | 2.87 (1.60, 5.15) | 4.28E-04 |
| 7 | rs216743 | A | 0.10 | 2.42 (1.24, 4.75) | 9.84E-03 | 0.10 | 4.20 (1.72, 10.28) | 1.64E-03 |
| 7 | rs216744 | G | 0.10 | 2.42 (1.24 , 4.75) | 9.84E-03 | 0.10 | 4.20 (1.72, 10.28) | 1.64E-03 |
| 22 | rs131817 | T | 0.23 | 0.46 (0.24, 0.89) | 2.17E-02 | 0.23 | 0.52 (0.26, 1.03) | 6.12E-02 |
| 7 | rs4722999 | C | 0.32 | 1.83 (1.08, 3.10) | 2.42E-02 | 0.32 | 1.87 (1.02, 3.43) | 4.37E-02 |
| 17 | rs8069770 | T | 0.14 | 0.36 (0.15, 0.88) | 2.46E-02 | 0.14 | 0.40 (0.16, 1.00) | 4.88E-02 |
| 5 | rs6884962 | G | 0.49 | 1.88 (1.16, 3.04) | 1.05E-02 | 0.49 | 1.73 (1.02, 2.94) | 4.37E-02 |
| 12 | rs12579934 | T | 0.45 | 1.97 (1.19, 3.26) | 8.72E-03 | 0.45 | 5.12 (2.51, 10.46) | 7.54E-06 |
| 9 | rs12376718 | T | 0.14 | 1.78 (0.98, 3.22) | 5.74E-02 | 0.14 | 3.40 (1.67, 6.93) | 7.67E-04 |
| 16 | rs6540013 | G | 0.38 | 0.56 (0.34, 0.94) | 2.89E-02 | 0.38 | 0.61 (0.35, 1.08) | 8.77E-02 |
| 16 | rs12598821 | T | 0.48 | 0.76 (0.47, 1.24) | 2.72E-01 | 0.48 | 0.32 (0.17, 0.62) | 5.81E-04 |
| 1 | rs3861824 | A | 0.11 | 0.30 (0.10, 0.86) | 2.52E-02 | 0.11 | 0.30 (0.09, 0.97) | 4.41E-02 |

† Effect estimates and *p*-values for intrauterine and intrapartum HIV transmission phenotypes, summarized for the 20 most-significant SNPs from cumulative HIV MTCT analyses. CHR: Chromosome, A1: risk allele designated by *PLINK*, MAF: Minor Allele Frequency, OR: Odds Ratio, 95% CI: 95% Confidence Interval of the OR, *p*: adjusted by maternal HIV viral load *p*-value.
