# Supplementary material for: A whole genome association study of mother-to-child transmission of HIV in Malawi
Source: Genome Med. 2010 Mar 1;2(3):17. doi: 10.1186/gm138 (PMC2873795; doi:10.1186/gm138)
Supplement: Additional file 2 — A Word document giving effect estimates for SNPs near or within genes associated with HIV/AIDS. The data provided represent the genome-wide association analysis for specific regions that have previously demonstrated association with HIV/AIDS, described in the Introduction section. [file gm138-S2.DOCX]

Additional file 2. Effect estimates for SNPs near or within genes associated with HIV/AIDS

|  |  |  |  |  |  | **Cumulative HIV MTCT** | | | **Intrauterine Transmission** | | | **Intrapartum Transmission** | | |
| --- | --- | --- | --- | --- | --- | --- | --- | --- | --- | --- | --- | --- | --- | --- |
| **Within 10KB**  **of Gene:** | **CHR** | **SNP** | **BP** | **Type** | **A1** | **MAF** | **OR** | ***p*** | **MAF** | **OR** | ***p*** | **MAF** | **OR** | ***p*** |
| *SDC3* | 1 | rs6425688 | 31002692 | b | C | 0.10 | 1.45 | 2.13E-01 | 0.11 | 0.86 | 6.93E-01 | 0.11 | 2.11 | 5.22E-02 |
| *SDC3* | 1 | rs2796208 | 31004472 | b | C | 0.08 | 0.97 | 9.38E-01 | 0.08 | 1.15 | 7.49E-01 | 0.08 | 0.79 | 6.62E-01 |
| *SDC3* | 1 | rs2506969 | 31005532 | b | C | 0.10 | 0.90 | 7.34E-01 | 0.10 | 1.17 | 6.85E-01 | 0.10 | 0.80 | 6.56E-01 |
| *SDC3* | 1 | rs12408228 | 31006510 | b | A | 0.29 | 1.03 | 8.85E-01 | 0.28 | 1.01 | 9.64E-01 | 0.28 | 0.92 | 7.50E-01 |
| *SDC3* | 1 | rs4949320 | 31008663 | b | T | 0.33 | 1.07 | 7.49E-01 | 0.33 | 1.28 | 3.23E-01 | 0.33 | 0.80 | 4.60E-01 |
| *SDC3* | 1 | rs3766286 | 31013343 | a | C | 0.37 | 1.09 | 6.59E-01 | 0.37 | 1.04 | 8.92E-01 | 0.37 | 0.75 | 3.57E-01 |
| *SDC3* | 1 | rs2488238 | 31014045 | a | A | 0.15 | 1.00 | 9.88E-01 | 0.15 | 1.34 | 3.86E-01 | 0.15 | 0.69 | 3.93E-01 |
| *SDC3* | 1 | rs3820088 | 31014840 | a | A | 0.32 | 1.08 | 6.89E-01 | 0.32 | 1.19 | 4.89E-01 | 0.32 | 0.71 | 2.83E-01 |
| *SDC3* | 1 | rs2282440 | 31016413 | e | T | 0.02 | 0.79 | 7.49E-01 | 0.02 | 0.59 | 6.32E-01 | 0.02 | 0.59 | 6.37E-01 |
| *SDC3* | 1 | rs4949184 | 31016492 | e | A | 0.34 | 1.10 | 6.49E-01 | 0.34 | 1.02 | 9.46E-01 | 0.34 | 0.75 | 3.56E-01 |
| *SDC3* | 1 | rs2491133 | 31019659 | a | T | 0.08 | 0.42 | 3.12E-02 | 0.08 | 0.68 | 4.37E-01 | 0.08 | 0.34 | 8.96E-02 |
| *SDC3* | 1 | rs1891419 | 31020606 | h | A | 0.05 | 0.85 | 7.39E-01 | 0.05 | 0.93 | 9.06E-01 | 0.05 | 0.56 | 4.69E-01 |
| *SDC3* | 1 | rs6695189 | 31023170 | d | A | 0.03 | 2.02 | 1.68E-01 | 0.04 | 0.94 | 9.23E-01 | 0.04 | 3.67 | 2.85E-02 |
| *SDC3* | 1 | rs16834123 | 31023257 | d | C | 0.26 | 0.82 | 3.96E-01 | 0.26 | 0.64 | 1.44E-01 | 0.26 | 0.90 | 7.35E-01 |
| *SDC3* | 1 | rs10753239 | 31031317 | d | T | 0.08 | 0.54 | 9.68E-02 | 0.08 | 0.73 | 5.04E-01 | 0.08 | 0.58 | 2.87E-01 |
| *SDC3* | 1 | rs7529390 | 31041779 | d | T | 0.12 | 0.85 | 5.91E-01 | 0.12 | 1.40 | 3.38E-01 | 0.12 | 0.82 | 6.60E-01 |
| *SDC3* | 1 | rs12085929 | 31041827 | d | C | 0.43 | 1.00 | 9.80E-01 | 0.44 | 0.89 | 6.27E-01 | 0.44 | 1.28 | 3.61E-01 |
| *SDC3* | 1 | rs11810325 | 31044662 | d | A | 0.04 | 1.56 | 3.41E-01 | 0.04 | 0.82 | 7.58E-01 | 0.04 | 2.79 | 5.88E-02 |
| *SDC3* | 1 | rs10158813 | 31047379 | d | C | 0.13 | 0.67 | 1.80E-01 | 0.13 | 0.57 | 1.75E-01 | 0.13 | 0.67 | 3.46E-01 |
| *SDC3* | 1 | rs6680835 | 31056423 | c | A | 0.21 | 0.95 | 8.30E-01 | 0.21 | 1.60 | 9.17E-02 | 0.21 | 0.44 | 5.11E-02 |
| *SDC3* | 1 | rs12097621 | 31056449 | c | C | 0.15 | 1.13 | 6.40E-01 | 0.15 | 1.15 | 6.66E-01 | 0.15 | 1.00 | 9.89E-01 |
| *SDC3* | 1 | rs6425689 | 31056811 | c | C | 0.05 | 1.86 | 1.42E-01 | 0.05 | 1.02 | 9.70E-01 | 0.05 | 3.40 | 2.18E-02 |
| *SDC3* | 1 | rs10914232 | 31057652 | c | C | 0.44 | 0.76 | 1.63E-01 | 0.44 | 0.65 | 7.97E-02 | 0.44 | 0.88 | 6.41E-01 |
| *SDC1* | 2 | rs2881925 | 20312322 | c | A | 0.25 | 0.99 | 9.59E-01 | 0.25 | 1.26 | 4.17E-01 | 0.25 | 0.85 | 6.43E-01 |
| *SDC1* | 2 | rs2881926 | 20313481 | c | A | 0.30 | 1.04 | 8.48E-01 | 0.30 | 1.42 | 1.83E-01 | 0.30 | 0.90 | 7.29E-01 |
| *SDC1* | 2 | rs6749689 | 20317750 | b | C | 0.41 | 1.05 | 7.91E-01 | 0.41 | 0.88 | 5.99E-01 | 0.41 | 1.24 | 4.22E-01 |
| *SDC1* | 2 | rs2348476 | 20321684 | b | T | 0.08 | 0.72 | 3.87E-01 | 0.08 | 0.82 | 6.81E-01 | 0.08 | 1.04 | 9.45E-01 |
| *SDC1* | 2 | rs4432408 | 20321928 | b | A | 0.34 | 1.05 | 8.26E-01 | 0.34 | 1.04 | 8.93E-01 | 0.34 | 0.93 | 8.20E-01 |
| *SDC1* | 2 | rs3771254 | 20327873 | d | A | 0.11 | 0.90 | 7.46E-01 | 0.11 | 0.91 | 8.17E-01 | 0.11 | 0.68 | 4.40E-01 |
| *SDC1* | 2 | rs2015110 | 20330950 | d | A | 0.06 | 0.80 | 6.14E-01 | 0.05 | 0.85 | 7.77E-01 | 0.05 | 0.96 | 9.46E-01 |
| *SDC1* | 2 | rs3771240 | 20334314 | d | G | 0.43 | 1.30 | 2.14E-01 | 0.42 | 1.18 | 5.31E-01 | 0.42 | 1.10 | 7.33E-01 |
| *SDC1* | 2 | rs1106111 | 20340740 | d | T | 0.24 | 1.57 | 4.80E-02 | 0.24 | 1.51 | 1.33E-01 | 0.24 | 1.39 | 3.04E-01 |
| *CXCR4* | 2 | rs16832731 | 136698435 | c | G | 0.49 | 1.17 | 4.67E-01 | 0.49 | 1.41 | 2.10E-01 | 0.49 | 1.21 | 5.25E-01 |
| *CXCR4* | 2 | rs16832740 | 136701608 | b | C | 0.19 | 0.59 | 4.87E-02 | 0.19 | 0.41 | 2.14E-02 | 0.19 | 1.07 | 8.53E-01 |
| *CXCR4* | 2 | rs4954391 | 136717555 | c | C | 0.45 | 0.73 | 1.26E-01 | 0.45 | 0.66 | 1.14E-01 | 0.45 | 0.69 | 1.84E-01 |
| *CXCR4* | 2 | rs10191360 | 136718411 | c | C | 0.08 | 1.26 | 5.34E-01 | 0.08 | 2.18 | 5.99E-02 | 0.08 | 0.80 | 7.20E-01 |
| *IL8* | 4 | rs4694636 | 74963844 | c | T | 0.13 | 0.94 | 8.32E-01 | 0.13 | 1.06 | 8.67E-01 | 0.13 | 1.31 | 4.95E-01 |
| *IL8* | 4 | rs16849934 | 74965708 | c | C | 0.12 | 0.92 | 7.68E-01 | 0.12 | 0.93 | 8.32E-01 | 0.12 | 0.88 | 7.61E-01 |
| *IL8* | 4 | rs2227538 | 74971410 | a | T | 0.24 | 0.94 | 7.81E-01 | 0.23 | 1.12 | 6.78E-01 | 0.23 | 1.04 | 9.12E-01 |
| *IL8* | 4 | rs1951699 | 74983944 | c | A | 0.18 | 1.12 | 6.25E-01 | 0.19 | 0.68 | 2.36E-01 | 0.19 | 1.06 | 8.67E-01 |
| *IL4* | 5 | rs2243206 | 132028964 | i | T | 0.13 | 0.77 | 4.11E-01 | 0.13 | 1.24 | 5.80E-01 | 0.13 | 0.27 | 2.48E-02 |
| *IL4* | 5 | rs2243220 | 132030660 | i | C | 0.05 | 0.63 | 3.08E-01 | 0.05 | 0.72 | 5.85E-01 | 0.05 | 0.88 | 8.42E-01 |
| *IL4* | 5 | rs2243300 | 132031985 | i | T | 0.02 | 2.08 | 3.24E-01 | 0.02 | 0.56 | 5.92E-01 | 0.02 | 4.44 | 1.11E-01 |
| *IL4* | 5 | rs2243248 | 132036543 | i | C | 0.21 | 0.75 | 2.38E-01 | 0.21 | 1.03 | 9.32E-01 | 0.21 | 0.60 | 1.74E-01 |
| *IL4* | 5 | rs2070874 | 132037609 | a | A | 0.49 | 1.18 | 3.92E-01 | 0.49 | 1.10 | 6.95E-01 | 0.49 | 1.79 | 3.39E-02 |
| *IL4* | 5 | rs2243268 | 132041862 | d | C | 0.34 | 1.08 | 6.87E-01 | 0.34 | 1.06 | 8.15E-01 | 0.34 | 1.45 | 1.98E-01 |
| *IL4* | 5 | rs2243279 | 132044126 | d | T | 0.08 | 0.66 | 2.55E-01 | 0.08 | 0.77 | 5.88E-01 | 0.08 | 0.92 | 8.57E-01 |
| *IL4* | 5 | rs2243288 | 132045843 | d | T | 0.32 | 1.44 | 5.84E-02 | 0.31 | 0.90 | 6.72E-01 | 0.31 | 1.38 | 2.27E-01 |
| *IL4* | 5 | rs2243290 | 132046068 | d | T | 0.40 | 0.97 | 8.50E-01 | 0.40 | 1.07 | 7.85E-01 | 0.40 | 1.20 | 4.77E-01 |
| *HLA-G* | 6 | rs1619379 | 29893214 | i | T | 0.41 | 0.89 | 5.34E-01 | 0.41 | 0.96 | 8.81E-01 | 0.41 | 1.00 | 9.94E-01 |
| *HLA-G* | 6 | rs2743933 | 29894199 | c | G | 0.10 | 0.90 | 7.50E-01 | 0.10 | 0.79 | 5.89E-01 | 0.10 | 0.52 | 2.74E-01 |
| *HLA-G* | 6 | rs2523790 | 29911629 | b | G | 0.49 | 1.19 | 3.58E-01 | 0.49 | 0.95 | 8.43E-01 | 0.49 | 1.34 | 2.67E-01 |
| *HLA-G* | 6 | rs2394180 | 29913178 | c | C | 0.27 | 1.27 | 2.32E-01 | 0.27 | 1.04 | 8.66E-01 | 0.27 | 1.34 | 2.84E-01 |
| *HLA-G* | 6 | rs9258525 | 29914320 | c | C | 0.06 | 0.82 | 6.52E-01 | 0.06 | 1.01 | 9.93E-01 | 0.06 | 1.50 | 4.87E-01 |
| *HLA-G* | 6 | rs2743937 | 29915902 | c | C | 0.49 | 1.22 | 2.77E-01 | 0.49 | 0.98 | 9.22E-01 | 0.49 | 1.39 | 2.09E-01 |
| *HLA-G* | 6 | rs2735007 | 29916178 | c | T | 0.49 | 1.22 | 2.77E-01 | 0.49 | 0.98 | 9.22E-01 | 0.49 | 1.39 | 2.09E-01 |
| *HLA-G* | 6 | rs2735003 | 29916613 | c | C | 0.49 | 1.23 | 2.76E-01 | 0.49 | 0.98 | 9.23E-01 | 0.49 | 1.39 | 2.09E-01 |
| *HLA-A* | 6 | rs2524005 | 30007656 | i | T | 0.10 | 0.99 | 9.79E-01 | 0.10 | 0.99 | 9.86E-01 | 0.10 | 0.49 | 1.57E-01 |
| *HLA-A* | 6 | rs2860580 | 30014670 | i | A | 0.48 | 0.59 | 1.18E-02 | 0.48 | 0.76 | 2.82E-01 | 0.48 | 0.37 | 3.22E-03 |
| *HLA-C* | 6 | rs3130542 | 31340090 | b | T | 0.17 | 1.18 | 5.21E-01 | 0.17 | 1.87 | 4.06E-02 | 0.17 | 0.48 | 1.25E-01 |
| *HLA-C* | 6 | rs2844623 | 31340522 | b | A | 0.16 | 0.74 | 2.74E-01 | 0.16 | 0.60 | 1.66E-01 | 0.16 | 0.97 | 9.35E-01 |
| *HLA-C* | 6 | rs9264508 | 31341193 | b | T | 0.17 | 1.20 | 4.87E-01 | 0.17 | 1.85 | 4.28E-02 | 0.17 | 0.47 | 1.19E-01 |
| *HLA-C* | 6 | rs9264532 | 31342360 | b | G | 0.37 | 0.79 | 2.30E-01 | 0.37 | 1.10 | 7.01E-01 | 0.37 | 0.58 | 8.52E-02 |
| *HLA-C* | 6 | rs2524099 | 31344030 | b | C | 0.32 | 1.06 | 7.70E-01 | 0.32 | 1.18 | 5.13E-01 | 0.32 | 1.02 | 9.56E-01 |
| *HLA-C* | 6 | rs9394047 | 31344229 | b | G | 0.07 | 1.29 | 4.57E-01 | 0.07 | 1.84 | 1.11E-01 | 0.07 | 0.98 | 9.70E-01 |
| *HLA-C* | 6 | rs2074488 | 31348410 | i | A | 0.10 | 0.99 | 9.81E-01 | 0.10 | 0.74 | 4.93E-01 | 0.10 | 1.35 | 4.51E-01 |
| *HLA-C* | 6 | rs2395471 | 31348671 | i | T | 0.45 | 1.22 | 3.39E-01 | 0.45 | 1.11 | 6.85E-01 | 0.45 | 1.40 | 2.62E-01 |
| *HLA-C* | 6 | rs2249742 | 31348700 | i | C | 0.47 | 1.07 | 7.19E-01 | 0.47 | 1.12 | 6.44E-01 | 0.47 | 1.01 | 9.84E-01 |
| *HLA-C* | 6 | rs5010528 | 31349011 | i | G | 0.18 | 1.69 | 3.99E-02 | 0.18 | 1.49 | 1.86E-01 | 0.18 | 1.22 | 5.75E-01 |
| *HLA-C* | 6 | rs13207315 | 31349106 | i | C | 0.12 | 0.83 | 5.33E-01 | 0.12 | 0.96 | 9.17E-01 | 0.12 | 1.18 | 6.77E-01 |
| *HLA-C* | 6 | rs12111032 | 31350170 | i | C | 0.24 | 0.88 | 5.66E-01 | 0.24 | 0.88 | 6.61E-01 | 0.24 | 1.01 | 9.85E-01 |
| *HLA-C* | 6 | rs9461680 | 31351326 | i | A | 0.26 | 0.62 | 4.04E-02 | 0.25 | 0.53 | 4.28E-02 | 0.25 | 0.73 | 3.30E-01 |
| *HLA-C* | 6 | rs2524077 | 31351582 | j | A | 0.16 | 0.74 | 2.48E-01 | 0.16 | 0.73 | 3.57E-01 | 0.16 | 0.65 | 2.82E-01 |
| *HLA-C* | 6 | rs3130696 | 31351863 | j | A | 0.19 | 1.92 | 7.75E-03 | 0.19 | 1.93 | 1.67E-02 | 0.19 | 1.50 | 2.19E-01 |
| *HLA-C* | 6 | rs2524073 | 31352214 | j | A | 0.14 | 0.59 | 7.14E-02 | 0.14 | 0.43 | 5.48E-02 | 0.14 | 0.58 | 2.06E-01 |
| *HLA-C* | 6 | rs2524070 | 31352499 | j | A | 0.22 | 0.64 | 6.76E-02 | 0.22 | 0.57 | 8.50E-02 | 0.22 | 0.79 | 4.76E-01 |
| *HLA-C* | 6 | rs6906846 | 31353715 | j | T | 0.29 | 1.33 | 1.69E-01 | 0.29 | 1.40 | 1.81E-01 | 0.29 | 1.19 | 5.54E-01 |
| *HLA-C* | 6 | rs7382297 | 31355046 | i | A | 0.03 | 0.83 | 7.39E-01 | 0.03 | 1.54 | 4.78E-01 | 0.03 | 0.42 | 4.31E-01 |
| *HLA-C* | 6 | rs4386816 | 31355114 | i | C | 0.23 | 1.43 | 1.29E-01 | 0.22 | 1.26 | 4.26E-01 | 0.22 | 0.97 | 9.22E-01 |
| *HLA-B* | 6 | rs3134792 | 31420305 | c | C | 0.05 | 1.31 | 5.53E-01 | 0.05 | 0.90 | 8.63E-01 | 0.05 | 2.21 | 1.41E-01 |
| *HLA-B* | 6 | rs2156875 | 31425326 | b | G | 0.41 | 0.78 | 2.03E-01 | 0.42 | 1.01 | 9.63E-01 | 0.42 | 0.85 | 5.50E-01 |
| *HLA-B* | 6 | rs2523619 | 31426123 | b | C | 0.27 | 0.82 | 3.51E-01 | 0.27 | 0.82 | 4.76E-01 | 0.27 | 1.02 | 9.47E-01 |
| *HLA-B* | 6 | rs2442719 | 31428517 | i | T | 0.44 | 0.83 | 3.25E-01 | 0.44 | 1.01 | 9.79E-01 | 0.44 | 0.86 | 5.80E-01 |
| *HLA-B* | 6 | rs2596503 | 31428789 | i | A | 0.14 | 0.62 | 9.63E-02 | 0.14 | 0.70 | 3.39E-01 | 0.14 | 0.59 | 2.25E-01 |
| *HLA-B* | 6 | rs2596501 | 31429190 | i | C | 0.39 | 0.74 | 1.50E-01 | 0.39 | 0.95 | 8.52E-01 | 0.39 | 0.87 | 6.23E-01 |
| *HLA-B* | 6 | rs1058026 | 31429664 | a | G | 0.19 | 1.43 | 1.65E-01 | 0.19 | 1.31 | 3.87E-01 | 0.19 | 1.46 | 2.93E-01 |
| *HLA-B* | 6 | rs2523608 | 31430538 | d | G | 0.38 | 0.66 | 4.44E-02 | 0.38 | 0.76 | 2.95E-01 | 0.38 | 0.67 | 1.75E-01 |
| *HLA-B* | 6 | rs2523589 | 31435313 | i | G | 0.35 | 0.64 | 2.47E-02 | 0.35 | 0.53 | 1.96E-02 | 0.35 | 0.81 | 4.50E-01 |
| *HLA-B* | 6 | rs2523554 | 31439808 | b | C | 0.16 | 0.87 | 5.72E-01 | 0.17 | 0.73 | 3.65E-01 | 0.17 | 1.12 | 7.52E-01 |
| *HLA-B* | 6 | rs2596551 | 31440218 | b | C | 0.19 | 1.58 | 7.66E-02 | 0.18 | 1.39 | 2.84E-01 | 0.18 | 1.22 | 6.09E-01 |
| *HLA-B* | 6 | rs2844575 | 31442924 | i | C | 0.49 | 0.94 | 7.55E-01 | 0.49 | 0.91 | 6.97E-01 | 0.49 | 0.94 | 8.20E-01 |
| *SDC2* | 8 | rs10808350 | 97565172 | c | T | 0.13 | 1.22 | 4.97E-01 | 0.13 | 0.57 | 1.91E-01 | 0.13 | 1.64 | 1.89E-01 |
| *SDC2* | 8 | rs2439516 | 97573917 | i | A | 0.13 | 1.20 | 5.39E-01 | 0.13 | 1.38 | 3.72E-01 | 0.13 | 0.95 | 9.13E-01 |
| *SDC2* | 8 | rs2437770 | 97574721 | i | T | 0.07 | 0.63 | 2.48E-01 | 0.07 | 0.44 | 1.91E-01 | 0.07 | 0.65 | 4.41E-01 |
| *SDC2* | 8 | rs1348563 | 97577537 | d | T | 0.40 | 1.04 | 8.38E-01 | 0.40 | 1.02 | 9.26E-01 | 0.40 | 1.39 | 2.38E-01 |
| *SDC2* | 8 | rs895033 | 97577731 | d | A | 0.24 | 0.76 | 2.28E-01 | 0.24 | 0.96 | 8.96E-01 | 0.24 | 0.89 | 7.08E-01 |
| *SDC2* | 8 | rs13270556 | 97579308 | d | C | 0.12 | 0.78 | 4.10E-01 | 0.13 | 0.64 | 2.83E-01 | 0.13 | 1.25 | 5.94E-01 |
| *SDC2* | 8 | rs12056723 | 97584217 | d | C | 0.12 | 1.02 | 9.46E-01 | 0.12 | 1.40 | 3.35E-01 | 0.12 | 0.46 | 1.36E-01 |
| *SDC2* | 8 | rs2582814 | 97586160 | d | C | 0.18 | 1.79 | 2.04E-02 | 0.18 | 1.12 | 7.12E-01 | 0.18 | 2.16 | 2.64E-02 |
| *SDC2* | 8 | rs2439518 | 97586565 | d | C | 0.39 | 1.59 | 2.27E-02 | 0.39 | 1.12 | 6.66E-01 | 0.39 | 1.45 | 1.89E-01 |
| *SDC2* | 8 | rs2008026 | 97587263 | d | G | 0.15 | 0.69 | 1.82E-01 | 0.15 | 0.59 | 1.67E-01 | 0.15 | 0.98 | 9.48E-01 |
| *SDC2* | 8 | rs2439520 | 97588101 | d | G | 0.25 | 1.13 | 5.83E-01 | 0.25 | 1.19 | 5.27E-01 | 0.25 | 0.89 | 7.08E-01 |
| *SDC2* | 8 | rs2589183 | 97591685 | d | T | 0.23 | 0.68 | 1.04E-01 | 0.23 | 0.84 | 5.59E-01 | 0.23 | 0.85 | 6.09E-01 |
| *SDC2* | 8 | rs2253255 | 97591719 | d | G | 0.19 | 0.96 | 8.53E-01 | 0.19 | 1.21 | 5.03E-01 | 0.19 | 0.67 | 2.73E-01 |
| *SDC2* | 8 | rs6985568 | 97591763 | d | A | 0.10 | 0.92 | 7.96E-01 | 0.10 | 0.75 | 5.11E-01 | 0.10 | 1.14 | 7.83E-01 |
| *SDC2* | 8 | rs2575738 | 97599578 | d | A | 0.30 | 0.87 | 5.45E-01 | 0.30 | 1.50 | 1.36E-01 | 0.30 | 0.46 | 2.44E-02 |
| *SDC2* | 8 | rs2582819 | 97600212 | d | G | 0.44 | 1.23 | 2.67E-01 | 0.44 | 0.97 | 8.90E-01 | 0.44 | 1.75 | 3.99E-02 |
| *SDC2* | 8 | rs2575735 | 97603827 | d | C | 0.37 | 0.88 | 5.28E-01 | 0.37 | 0.61 | 7.46E-02 | 0.37 | 0.97 | 9.13E-01 |
| *SDC2* | 8 | rs2582822 | 97603992 | d | C | 0.33 | 0.87 | 4.84E-01 | 0.33 | 1.39 | 1.82E-01 | 0.33 | 0.57 | 6.96E-02 |
| *SDC2* | 8 | rs2439523 | 97604209 | d | T | 0.05 | 1.02 | 9.69E-01 | 0.05 | 2.02 | 1.46E-01 | 0.05 | 0.37 | 3.29E-01 |
| *SDC2* | 8 | rs2439525 | 97606217 | d | C | 0.18 | 1.01 | 9.80E-01 | 0.18 | 1.85 | 3.47E-02 | 0.18 | 0.52 | 1.13E-01 |
| *SDC2* | 8 | rs7003874 | 97607128 | d | T | 0.16 | 0.91 | 7.15E-01 | 0.16 | 0.50 | 8.78E-02 | 0.16 | 1.61 | 1.70E-01 |
| *SDC2* | 8 | rs2575734 | 97607381 | d | C | 0.39 | 1.01 | 9.55E-01 | 0.39 | 0.92 | 7.32E-01 | 0.39 | 0.77 | 3.48E-01 |
| *SDC2* | 8 | rs2589212 | 97609783 | d | G | 0.22 | 1.19 | 4.58E-01 | 0.22 | 0.95 | 8.65E-01 | 0.22 | 1.78 | 7.04E-02 |
| *SDC2* | 8 | rs2589208 | 97611112 | d | T | 0.47 | 0.89 | 5.28E-01 | 0.47 | 0.96 | 8.76E-01 | 0.47 | 0.62 | 7.36E-02 |
| *SDC2* | 8 | rs895034 | 97612566 | d | C | 0.37 | 0.90 | 6.08E-01 | 0.38 | 0.92 | 7.52E-01 | 0.38 | 0.58 | 6.35E-02 |
| *SDC2* | 8 | rs2440681 | 97613757 | d | T | 0.40 | 1.06 | 7.91E-01 | 0.40 | 0.88 | 6.00E-01 | 0.40 | 1.42 | 2.43E-01 |
| *SDC2* | 8 | rs2437774 | 97616306 | d | T | 0.13 | 1.53 | 1.29E-01 | 0.13 | 1.81 | 6.59E-02 | 0.13 | 0.59 | 2.69E-01 |
| *SDC2* | 8 | rs2589203 | 97618876 | d | G | 0.29 | 0.68 | 8.93E-02 | 0.29 | 0.57 | 6.53E-02 | 0.29 | 0.89 | 7.04E-01 |
| *SDC2* | 8 | rs2589198 | 97625741 | d | C | 0.30 | 1.58 | 2.88E-02 | 0.30 | 1.54 | 8.36E-02 | 0.30 | 1.37 | 2.66E-01 |
| *SDC2* | 8 | rs2582840 | 97628016 | d | G | 0.21 | 1.32 | 2.52E-01 | 0.21 | 1.76 | 4.89E-02 | 0.21 | 1.03 | 9.26E-01 |
| *SDC2* | 8 | rs10100191 | 97628338 | d | G | 0.30 | 0.72 | 1.19E-01 | 0.30 | 0.90 | 6.97E-01 | 0.30 | 0.70 | 2.55E-01 |
| *SDC2* | 8 | rs16894717 | 97628841 | d | A | 0.16 | 1.21 | 4.80E-01 | 0.16 | 0.52 | 9.46E-02 | 0.16 | 2.06 | 4.31E-02 |
| *SDC2* | 8 | rs2582842 | 97632820 | d | C | 0.15 | 0.56 | 3.66E-02 | 0.15 | 0.63 | 2.17E-01 | 0.15 | 0.49 | 9.66E-02 |
| *SDC2* | 8 | rs1444572 | 97634058 | d | G | 0.18 | 0.69 | 1.52E-01 | 0.18 | 0.94 | 8.55E-01 | 0.18 | 0.47 | 6.89E-02 |
| *SDC2* | 8 | rs2582843 | 97635973 | d | G | 0.42 | 0.61 | 1.74E-02 | 0.42 | 0.83 | 4.47E-01 | 0.42 | 0.53 | 3.45E-02 |
| *SDC2* | 8 | rs1984456 | 97636282 | d | A | 0.19 | 0.75 | 2.45E-01 | 0.20 | 0.93 | 8.09E-01 | 0.20 | 0.67 | 2.97E-01 |
| *SDC2* | 8 | rs2016529 | 97647289 | d | G | 0.23 | 0.99 | 9.56E-01 | 0.23 | 0.35 | 4.98E-03 | 0.23 | 1.32 | 3.71E-01 |
| *SDC2* | 8 | rs7831863 | 97652665 | d | A | 0.28 | 1.04 | 8.68E-01 | 0.28 | 1.11 | 6.88E-01 | 0.28 | 0.99 | 9.85E-01 |
| *SDC2* | 8 | rs2575710 | 97653152 | d | T | 0.48 | 0.93 | 7.28E-01 | 0.49 | 1.12 | 6.51E-01 | 0.49 | 0.70 | 2.10E-01 |
| *SDC2* | 8 | rs2514781 | 97655328 | d | T | 0.17 | 1.09 | 7.46E-01 | 0.16 | 1.14 | 6.91E-01 | 0.16 | 1.38 | 4.19E-01 |
| *SDC2* | 8 | rs2464474 | 97666848 | d | C | 0.30 | 0.90 | 6.41E-01 | 0.30 | 0.79 | 3.93E-01 | 0.30 | 1.14 | 6.86E-01 |
| *SDC2* | 8 | rs16894821 | 97673288 | d | C | 0.18 | 1.03 | 8.93E-01 | 0.19 | 0.75 | 3.80E-01 | 0.19 | 0.88 | 7.16E-01 |
| *SDC2* | 8 | rs1126681 | 97674976 | h | T | 0.04 | 0.31 | 4.54E-02 | 0.04 | 0.21 | 1.36E-01 | 0.04 | 0.43 | 2.82E-01 |
| *SDC2* | 8 | rs2704256 | 97676598 | d | G | 0.04 | 0.31 | 4.54E-02 | 0.04 | 0.21 | 1.36E-01 | 0.04 | 0.43 | 2.82E-01 |
| *SDC2* | 8 | rs16892175 | 97677038 | d | C | 0.09 | 1.37 | 3.37E-01 | 0.09 | 1.03 | 9.42E-01 | 0.09 | 0.68 | 4.57E-01 |
| *SDC2* | 8 | rs2455049 | 97677281 | d | C | 0.17 | 1.29 | 3.44E-01 | 0.16 | 1.24 | 5.24E-01 | 0.16 | 2.06 | 7.64E-02 |
| *SDC2* | 8 | rs2704271 | 97677570 | d | C | 0.33 | 0.84 | 3.81E-01 | 0.33 | 0.68 | 1.44E-01 | 0.33 | 0.69 | 1.93E-01 |
| *SDC2* | 8 | rs16894859 | 97679264 | d | G | 0.36 | 0.86 | 4.59E-01 | 0.36 | 1.07 | 7.98E-01 | 0.36 | 0.64 | 1.46E-01 |
| *SDC2* | 8 | rs2582801 | 97680069 | d | C | 0.26 | 1.16 | 5.09E-01 | 0.26 | 1.19 | 5.12E-01 | 0.26 | 1.34 | 3.34E-01 |
| *SDC2* | 8 | rs1561158 | 97684411 | d | A | 0.09 | 0.75 | 3.86E-01 | 0.10 | 0.42 | 1.09E-01 | 0.10 | 1.13 | 7.62E-01 |
| *SDC2* | 8 | rs2575741 | 97688180 | d | A | 0.07 | 0.67 | 3.10E-01 | 0.08 | 0.98 | 9.60E-01 | 0.08 | 0.47 | 2.32E-01 |
| *SDC2* | 8 | rs2651458 | 97694626 | b | C | 0.21 | 1.06 | 7.98E-01 | 0.22 | 1.04 | 8.94E-01 | 0.22 | 1.39 | 3.10E-01 |
| *SDC2* | 8 | rs10955078 | 97699082 | c | C | 0.24 | 1.23 | 3.56E-01 | 0.24 | 1.62 | 7.14E-02 | 0.24 | 1.18 | 6.27E-01 |
| *SDC2* | 8 | rs10090346 | 97699705 | c | A | 0.45 | 1.16 | 4.37E-01 | 0.46 | 0.90 | 6.49E-01 | 0.46 | 1.47 | 1.59E-01 |
| *SDC2* | 8 | rs714046 | 97700749 | c | A | 0.25 | 0.67 | 8.05E-02 | 0.25 | 1.10 | 7.26E-01 | 0.25 | 0.39 | 1.50E-02 |
| *CXCL12* | 10 | rs266105 | 44175669 | c | A | 0.21 | 1.52 | 6.88E-02 | 0.22 | 1.62 | 7.17E-02 | 0.22 | 1.39 | 3.50E-01 |
| *CXCL12* | 10 | rs10900029 | 44182226 | b | T | 0.12 | 1.04 | 8.94E-01 | 0.12 | 0.58 | 1.98E-01 | 0.12 | 1.36 | 4.55E-01 |
| *CXCL12* | 10 | rs266094 | 44184306 | b | A | 0.26 | 1.33 | 1.82E-01 | 0.27 | 1.60 | 6.90E-02 | 0.27 | 0.89 | 7.19E-01 |
| *CXCL12* | 10 | rs1065297 | 44185982 | a | G | 0.10 | 1.37 | 3.20E-01 | 0.10 | 1.62 | 1.88E-01 | 0.10 | 1.47 | 3.90E-01 |
| *CXCL12* | 10 | rs197452 | 44190246 | d | T | 0.10 | 0.99 | 9.77E-01 | 0.10 | 0.95 | 8.89E-01 | 0.10 | 1.18 | 6.93E-01 |
| *CXCL12* | 10 | rs266087 | 44191068 | d | A | 0.26 | 0.79 | 3.00E-01 | 0.25 | 0.49 | 3.18E-02 | 0.25 | 1.04 | 9.06E-01 |
| *CXCL12* | 10 | rs2297630 | 44191554 | d | T | 0.01 | 0.33 | 3.21E-01 | 0.01 | 0.00 | 9.99E-01 | 0.01 | 1.16 | 9.03E-01 |
| *CXCL12* | 10 | rs266086 | 44191839 | d | A | 0.10 | 1.61 | 1.40E-01 | 0.10 | 1.91 | 6.49E-02 | 0.10 | 1.29 | 5.81E-01 |
| *CXCL12* | 10 | rs4948878 | 44194827 | d | C | 0.04 | 1.95 | 1.48E-01 | 0.04 | 2.26 | 8.91E-02 | 0.04 | 2.39 | 1.65E-01 |
| *CXCL12* | 10 | rs2839692 | 44194873 | d | G | 0.20 | 0.87 | 5.63E-01 | 0.20 | 1.00 | 9.93E-01 | 0.20 | 0.73 | 4.05E-01 |
| *CXCL12* | 10 | rs2839690 | 44195172 | d | C | 0.20 | 0.87 | 5.63E-01 | 0.20 | 1.00 | 9.93E-01 | 0.20 | 0.73 | 4.05E-01 |
| *CXCL12* | 10 | rs11592974 | 44197507 | d | C | 0.10 | 0.84 | 5.84E-01 | 0.10 | 0.86 | 7.22E-01 | 0.10 | 0.90 | 8.22E-01 |
| *CXCL12* | 10 | rs3780891 | 44198719 | f | A | 0.02 | 0.17 | 9.83E-02 | 0.02 | 0.55 | 5.82E-01 | 0.02 | 0.00 | 9.98E-01 |
| *CXCL12* | 10 | rs2861442 | 44204965 | i | G | 0.20 | 0.67 | 1.05E-01 | 0.20 | 0.84 | 5.66E-01 | 0.20 | 0.63 | 2.12E-01 |
| *CXCL12* | 10 | rs1855531 | 44209658 | c | A | 0.04 | 0.90 | 8.20E-01 | 0.04 | 0.76 | 6.58E-01 | 0.04 | 2.07 | 2.31E-01 |
| *MBL2* | 10 | rs3829168 | 54185596 | i | G | 0.13 | 1.23 | 4.76E-01 | 0.13 | 0.83 | 6.15E-01 | 0.13 | 1.05 | 8.97E-01 |
| *MBL2* | 10 | rs10508975 | 54186337 | i | G | 0.06 | 0.96 | 9.09E-01 | 0.06 | 1.30 | 5.66E-01 | 0.06 | 0.80 | 7.18E-01 |
| *MBL2* | 10 | rs7098284 | 54187678 | i | C | 0.34 | 1.17 | 4.31E-01 | 0.33 | 1.02 | 9.47E-01 | 0.33 | 1.35 | 2.80E-01 |
| *MBL2* | 10 | rs10740519 | 54187693 | i | T | 0.43 | 0.89 | 5.29E-01 | 0.44 | 1.06 | 8.08E-01 | 0.44 | 0.74 | 2.79E-01 |
| *MBL2* | 10 | rs920727 | 54188974 | i | A | 0.31 | 0.98 | 9.21E-01 | 0.31 | 1.25 | 3.56E-01 | 0.31 | 0.78 | 3.90E-01 |
| *MBL2* | 10 | rs2099902 | 54195855 | a | A | 0.37 | 0.83 | 3.18E-01 | 0.37 | 1.09 | 7.12E-01 | 0.37 | 0.77 | 3.34E-01 |
| *MBL2* | 10 | rs2120132 | 54196046 | a | C | 0.46 | 1.24 | 2.48E-01 | 0.45 | 0.95 | 8.25E-01 | 0.45 | 1.28 | 3.50E-01 |
| *MBL2* | 10 | rs10824792 | 54196212 | a | T | 0.10 | 0.95 | 8.60E-01 | 0.10 | 1.02 | 9.59E-01 | 0.10 | 0.72 | 5.00E-01 |
| *MBL2* | 10 | rs10082466 | 54196628 | a | C | 0.47 | 1.33 | 1.24E-01 | 0.46 | 1.07 | 7.73E-01 | 0.46 | 1.35 | 2.57E-01 |
| *MBL2* | 10 | rs1838065 | 54199263 | d | C | 0.06 | 1.18 | 6.63E-01 | 0.06 | 1.23 | 6.53E-01 | 0.06 | 0.87 | 7.93E-01 |
| *MBL2* | 10 | rs4935047 | 54200073 | d | C | 0.23 | 1.05 | 8.11E-01 | 0.23 | 1.00 | 9.97E-01 | 0.23 | 0.80 | 4.96E-01 |
| *MBL2* | 10 | rs10824796 | 54204619 | i | A | 0.26 | 0.86 | 4.75E-01 | 0.26 | 0.92 | 7.50E-01 | 0.26 | 0.89 | 6.93E-01 |
| *MBL2* | 10 | rs930506 | 54206557 | c | A | 0.43 | 1.11 | 5.68E-01 | 0.44 | 0.94 | 7.92E-01 | 0.44 | 0.98 | 9.31E-01 |
| *MBL2* | 10 | rs7899547 | 54206845 | c | T | 0.13 | 1.21 | 5.04E-01 | 0.14 | 0.92 | 8.21E-01 | 0.14 | 1.20 | 6.10E-01 |
| *MBL2* | 10 | rs11003132 | 54207453 | c | A | 0.07 | 1.19 | 6.46E-01 | 0.07 | 1.25 | 6.16E-01 | 0.07 | 0.32 | 1.35E-01 |
| *MBL2* | 10 | rs11003134 | 54208165 | c | T | 0.11 | 0.65 | 1.68E-01 | 0.11 | 0.81 | 5.96E-01 | 0.11 | 0.80 | 6.47E-01 |
| *HS3ST3A1* | 17 | rs16947982 | 13330666 | c | G | 0.07 | 1.23 | 5.63E-01 | 0.07 | 1.17 | 7.13E-01 | 0.07 | 1.13 | 8.02E-01 |
| *HS3ST3A1* | 17 | rs16947988 | 13332447 | c | T | 0.06 | 1.36 | 4.20E-01 | 0.06 | 1.23 | 6.55E-01 | 0.06 | 1.59 | 3.66E-01 |
| *HS3ST3A1* | 17 | rs7209938 | 13334607 | c | G | 0.21 | 0.79 | 3.05E-01 | 0.21 | 1.08 | 7.83E-01 | 0.21 | 0.92 | 7.92E-01 |
| *HS3ST3A1* | 17 | rs7216489 | 13336042 | b | T | 0.16 | 1.07 | 7.92E-01 | 0.16 | 1.48 | 2.01E-01 | 0.16 | 0.78 | 5.56E-01 |
| *HS3ST3A1* | 17 | rs6502266 | 13336445 | b | C | 0.04 | 0.42 | 1.18E-01 | 0.04 | 0.45 | 3.00E-01 | 0.04 | 0.22 | 1.47E-01 |
| *HS3ST3A1* | 17 | rs11078161 | 13336769 | b | G | 0.32 | 1.08 | 7.05E-01 | 0.32 | 1.03 | 9.03E-01 | 0.32 | 0.87 | 6.20E-01 |
| *HS3ST3A1* | 17 | rs9909849 | 13339585 | b | C | 0.13 | 0.62 | 1.03E-01 | 0.14 | 0.98 | 9.61E-01 | 0.14 | 0.37 | 5.73E-02 |
| *HS3ST3A1* | 17 | rs1029682 | 13344901 | d | A | 0.05 | 1.38 | 4.39E-01 | 0.05 | 1.84 | 1.92E-01 | 0.05 | 0.61 | 5.22E-01 |
| *HS3ST3A1* | 17 | rs6502269 | 13345506 | d | G | 0.05 | 1.38 | 4.39E-01 | 0.05 | 1.84 | 1.92E-01 | 0.05 | 0.61 | 5.22E-01 |
| *HS3ST3A1* | 17 | rs1860068 | 13348823 | d | G | 0.01 | 0.97 | 9.74E-01 | 0.01 | 1.14 | 9.10E-01 | 0.01 | 1.75 | 6.55E-01 |
| *HS3ST3A1* | 17 | rs3785710 | 13353836 | d | T | 0.11 | 1.61 | 1.20E-01 | 0.12 | 1.04 | 9.25E-01 | 0.12 | 1.68 | 1.88E-01 |
| *HS3ST3A1* | 17 | rs8073109 | 13366148 | d | C | 0.47 | 1.09 | 6.58E-01 | 0.47 | 1.13 | 6.09E-01 | 0.47 | 0.79 | 4.03E-01 |
| *HS3ST3A1* | 17 | rs9896557 | 13376273 | d | C | 0.05 | 0.36 | 4.76E-02 | 0.05 | 0.00 | 9.97E-01 | 0.05 | 0.86 | 8.05E-01 |
| *HS3ST3A1* | 17 | rs9897001 | 13376457 | d | A | 0.32 | 0.84 | 4.08E-01 | 0.31 | 0.72 | 2.17E-01 | 0.31 | 1.40 | 2.46E-01 |
| *HS3ST3A1* | 17 | rs11651136 | 13376656 | d | G | 0.29 | 0.87 | 5.05E-01 | 0.29 | 0.77 | 3.40E-01 | 0.29 | 1.42 | 2.19E-01 |
| *HS3ST3A1* | 17 | rs12941565 | 13383091 | d | T | 0.22 | 1.13 | 5.80E-01 | 0.23 | 1.29 | 3.28E-01 | 0.23 | 0.75 | 3.79E-01 |
| *HS3ST3A1* | 17 | rs9916623 | 13384523 | d | C | 0.46 | 0.88 | 5.20E-01 | 0.46 | 0.90 | 6.74E-01 | 0.46 | 1.21 | 4.94E-01 |
| *HS3ST3A1* | 17 | rs7224721 | 13385098 | d | G | 0.38 | 1.21 | 3.27E-01 | 0.38 | 1.29 | 2.83E-01 | 0.38 | 0.91 | 7.41E-01 |
| *HS3ST3A1* | 17 | rs16948063 | 13385406 | d | G | 0.09 | 1.08 | 8.24E-01 | 0.09 | 0.66 | 3.69E-01 | 0.09 | 0.50 | 2.00E-01 |
| *HS3ST3A1* | 17 | rs8082242 | 13389031 | d | C | 0.02 | 0.81 | 7.56E-01 | 0.02 | 0.64 | 6.57E-01 | 0.02 | 1.18 | 8.28E-01 |
| *HS3ST3A1* | 17 | rs3785705 | 13390698 | d | C | 0.02 | 0.81 | 7.56E-01 | 0.02 | 0.64 | 6.57E-01 | 0.02 | 1.18 | 8.28E-01 |
| *HS3ST3A1* | 17 | rs10521225 | 13392090 | d | C | 0.21 | 1.23 | 3.65E-01 | 0.21 | 1.43 | 1.87E-01 | 0.21 | 0.76 | 4.20E-01 |
| *HS3ST3A1* | 17 | rs9941385 | 13392537 | d | G | 0.03 | 0.48 | 2.29E-01 | 0.03 | 0.00 | 9.98E-01 | 0.03 | 1.03 | 9.70E-01 |
| *HS3ST3A1* | 17 | rs10521226 | 13392554 | d | A | 0.35 | 0.84 | 3.52E-01 | 0.35 | 0.64 | 7.86E-02 | 0.35 | 1.56 | 9.81E-02 |
| *HS3ST3A1* | 17 | rs1981629 | 13393581 | d | A | 0.47 | 0.70 | 5.95E-02 | 0.46 | 0.55 | 1.60E-02 | 0.46 | 1.55 | 1.08E-01 |
| *HS3ST3A1* | 17 | rs10521227 | 13394685 | d | T | 0.08 | 0.69 | 3.34E-01 | 0.08 | 0.32 | 7.79E-02 | 0.08 | 1.07 | 9.01E-01 |
| *HS3ST3A1* | 17 | rs3785702 | 13395760 | d | G | 0.41 | 0.89 | 5.48E-01 | 0.41 | 0.64 | 7.05E-02 | 0.41 | 1.22 | 4.55E-01 |
| *HS3ST3A1* | 17 | rs9303081 | 13397446 | d | T | 0.32 | 1.02 | 9.20E-01 | 0.32 | 0.58 | 4.86E-02 | 0.32 | 1.34 | 2.96E-01 |
| *HS3ST3A1* | 17 | rs7212673 | 13398971 | d | T | 0.31 | 0.98 | 9.18E-01 | 0.30 | 0.51 | 2.02E-02 | 0.30 | 1.30 | 3.54E-01 |
| *HS3ST3A1* | 17 | rs6502282 | 13399486 | d | T | 0.17 | 1.09 | 7.09E-01 | 0.17 | 0.52 | 7.02E-02 | 0.17 | 2.44 | 7.76E-03 |
| *HS3ST3A1* | 17 | rs3785697 | 13404912 | d | C | 0.49 | 1.21 | 3.26E-01 | 0.49 | 1.04 | 8.71E-01 | 0.49 | 1.04 | 8.81E-01 |
| *HS3ST3A1* | 17 | rs4399565 | 13407619 | d | C | 0.48 | 0.97 | 8.70E-01 | 0.48 | 1.43 | 1.52E-01 | 0.48 | 0.83 | 4.94E-01 |
| *HS3ST3A1* | 17 | rs2098026 | 13411022 | d | A | 0.35 | 1.05 | 7.98E-01 | 0.35 | 1.72 | 3.24E-02 | 0.35 | 0.78 | 4.07E-01 |
| *HS3ST3A1* | 17 | rs4588021 | 13415472 | d | T | 0.10 | 1.61 | 1.50E-01 | 0.09 | 0.74 | 5.12E-01 | 0.09 | 0.97 | 9.44E-01 |
| *HS3ST3A1* | 17 | rs3785692 | 13417128 | d | A | 0.34 | 1.33 | 1.54E-01 | 0.34 | 1.37 | 2.08E-01 | 0.34 | 1.35 | 2.53E-01 |
| *HS3ST3A1* | 17 | rs16948136 | 13417625 | d | A | 0.49 | 1.23 | 2.63E-01 | 0.50 | 1.34 | 2.18E-01 | 0.50 | 1.19 | 4.97E-01 |
| *HS3ST3A1* | 17 | rs8082581 | 13422178 | d | C | 0.30 | 0.91 | 6.63E-01 | 0.30 | 1.32 | 2.99E-01 | 0.30 | 0.74 | 3.37E-01 |
| *HS3ST3A1* | 17 | rs12952904 | 13427823 | d | A | 0.06 | 1.08 | 8.63E-01 | 0.05 | 0.89 | 8.41E-01 | 0.05 | 0.66 | 5.69E-01 |
| *HS3ST3A1* | 17 | rs11078168 | 13430337 | d | A | 0.29 | 0.68 | 7.95E-02 | 0.29 | 0.52 | 3.11E-02 | 0.29 | 0.54 | 5.79E-02 |
| *HS3ST3A1* | 17 | rs3785690 | 13436474 | d | T | 0.15 | 1.04 | 8.68E-01 | 0.15 | 0.48 | 6.35E-02 | 0.15 | 1.18 | 6.27E-01 |
| *HS3ST3A1* | 17 | rs726799 | 13441121 | d | T | 0.42 | 1.12 | 5.51E-01 | 0.41 | 1.05 | 8.26E-01 | 0.41 | 0.93 | 7.68E-01 |
| *HS3ST3A1* | 17 | rs8074072 | 13448594 | i | G | 0.23 | 1.12 | 6.15E-01 | 0.23 | 1.03 | 9.12E-01 | 0.23 | 1.28 | 4.22E-01 |
| *CCL5* | 17 | rs2291299 | 31215519 | d | C | 0.49 | 1.02 | 9.07E-01 | 0.49 | 1.07 | 7.77E-01 | 0.49 | 0.87 | 5.91E-01 |
| *CCL5* | 17 | rs16971600 | 31220991 | j | G | 0.07 | 0.70 | 3.05E-01 | 0.08 | 1.22 | 6.11E-01 | 0.08 | 0.54 | 3.12E-01 |
| *CCL5* | 17 | rs16963927 | 31228920 | d | C | 0.29 | 0.87 | 4.86E-01 | 0.29 | 0.78 | 3.58E-01 | 0.29 | 0.87 | 6.33E-01 |
| *CCL3* | 17 | rs11651881 | 31431019 | i | A | 0.20 | 1.27 | 2.99E-01 | 0.20 | 1.18 | 5.56E-01 | 0.20 | 1.11 | 7.39E-01 |
| *CCL3* | 17 | rs1851503 | 31436315 | j | C | 0.27 | 1.13 | 5.70E-01 | 0.27 | 0.97 | 8.98E-01 | 0.27 | 0.95 | 8.57E-01 |
| *CCL3* | 17 | rs9972960 | 31444192 | i | A | 0.35 | 1.18 | 4.06E-01 | 0.35 | 0.93 | 7.82E-01 | 0.35 | 1.19 | 5.48E-01 |
| *CCL3* | 17 | rs1634508 | 31450358 | i | T | 0.10 | 1.10 | 7.65E-01 | 0.10 | 1.74 | 1.06E-01 | 0.10 | 0.72 | 5.11E-01 |
| *CCL4* | 17 | rs1634508 | 31450358 | i | T | 0.10 | 1.10 | 7.65E-01 | 0.10 | 1.74 | 1.06E-01 | 0.10 | 0.72 | 5.11E-01 |
| *CCL4* | 17 | rs10491121 | 31454449 | i | T | 0.02 | 0.64 | 5.31E-01 | 0.02 | 0.51 | 5.31E-01 | 0.02 | 0.71 | 7.59E-01 |
| *CCL4* | 17 | rs1619600 | 31458788 | i | C | 0.10 | 0.94 | 8.39E-01 | 0.10 | 1.50 | 2.59E-01 | 0.10 | 0.63 | 3.61E-01 |
| *CCL4* | 17 | rs17617372 | 31458812 | i | A | 0.02 | 1.54 | 4.89E-01 | 0.02 | 1.57 | 5.22E-01 | 0.02 | 0.86 | 8.58E-01 |
| *CCL4* | 17 | rs1357365 | 31460645 | i | A | 0.39 | 1.01 | 9.61E-01 | 0.39 | 1.07 | 7.95E-01 | 0.39 | 1.22 | 5.01E-01 |
| *CCL3L1* | 17 | rs3744350 | 34515405 | d | A | 0.25 | 1.02 | 9.46E-01 | 0.25 | 0.73 | 2.72E-01 | 0.25 | 1.29 | 3.98E-01 |
| *CCL3L1* | 17 | rs3744351 | 34515507 | d | C | 0.28 | 1.22 | 3.54E-01 | 0.28 | 1.14 | 6.30E-01 | 0.28 | 1.32 | 3.56E-01 |
| *CCL3L1* | 17 | rs1989955 | 34515900 | d | G | 0.48 | 0.76 | 1.61E-01 | 0.48 | 0.68 | 1.20E-01 | 0.48 | 0.76 | 3.56E-01 |
| *CCL3L1* | 17 | rs16491 | 34521505 | d | T | 0.41 | 0.89 | 5.73E-01 | 0.41 | 0.67 | 1.26E-01 | 0.41 | 1.03 | 9.34E-01 |
| *CCL3L1* | 17 | rs3025160 | 34522004 | d | T | 0.38 | 0.87 | 4.61E-01 | 0.39 | 0.73 | 1.92E-01 | 0.39 | 1.40 | 1.89E-01 |
| *CCL3L1* | 17 | rs11871779 | 34523296 | d | A | 0.07 | 0.53 | 9.47E-02 | 0.07 | 0.59 | 3.26E-01 | 0.07 | 0.35 | 9.54E-02 |
| *CCL3L1* | 17 | rs16233 | 34524422 | d | C | 0.21 | 0.86 | 5.07E-01 | 0.21 | 0.50 | 4.36E-02 | 0.21 | 0.65 | 2.08E-01 |
| *CCL3L1* | 17 | rs16492 | 34524941 | d | C | 0.05 | 1.65 | 2.92E-01 | 0.04 | 1.43 | 5.13E-01 | 0.04 | 1.16 | 8.39E-01 |
| *CCL3L1* | 17 | rs739823 | 34526097 | d | T | 0.14 | 1.24 | 4.30E-01 | 0.14 | 1.36 | 3.55E-01 | 0.14 | 1.08 | 8.41E-01 |
| *CCL3L1* | 17 | rs3025166 | 34526415 | d | C | 0.01 | 0.99 | 9.91E-01 | 0.01 | 0.00 | 9.99E-01 | 0.01 | 0.93 | 9.53E-01 |
| *CCL3L1* | 17 | rs16235 | 34526657 | d | A | 0.20 | 1.35 | 2.33E-01 | 0.19 | 1.18 | 5.91E-01 | 0.19 | 1.27 | 4.88E-01 |
| *CCL3L1* | 17 | rs16238 | 34528576 | d | C | 0.48 | 1.26 | 2.47E-01 | 0.48 | 0.84 | 5.03E-01 | 0.48 | 1.10 | 7.51E-01 |
| *CCL3L1* | 17 | rs557570 | 34536855 | d | C | 0.27 | 1.63 | 3.02E-02 | 0.27 | 1.68 | 5.29E-02 | 0.27 | 1.20 | 5.59E-01 |
| *CCL3L1* | 17 | rs558317 | 34536896 | d | T | 0.11 | 0.71 | 2.71E-01 | 0.12 | 1.15 | 7.17E-01 | 0.12 | 0.80 | 6.15E-01 |
| *CCL3L1* | 17 | rs16495 | 34541657 | e | A | 0.10 | 1.24 | 4.84E-01 | 0.10 | 1.66 | 1.49E-01 | 0.10 | 0.86 | 7.38E-01 |
| *CCL3L1* | 17 | rs16502 | 34552020 | d | C | 0.08 | 0.87 | 7.17E-01 | 0.08 | 0.78 | 6.01E-01 | 0.08 | 1.10 | 8.48E-01 |
| *CCL3L1* | 17 | rs531728 | 34552287 | d | T | 0.05 | 0.97 | 9.47E-01 | 0.05 | 1.49 | 4.34E-01 | 0.05 | 1.04 | 9.57E-01 |
| *CCL3L1* | 17 | rs16505 | 34553903 | d | A | 0.49 | 0.93 | 7.08E-01 | 0.49 | 1.11 | 6.40E-01 | 0.49 | 0.87 | 6.10E-01 |
| *CCL3L1* | 17 | rs12946701 | 34556724 | d | C | 0.36 | 1.23 | 2.69E-01 | 0.36 | 1.41 | 1.40E-01 | 0.36 | 1.05 | 8.62E-01 |
| *CCL3L1* | 17 | rs16522 | 34570514 | e | G | 0.06 | 0.34 | 3.32E-02 | 0.05 | 0.18 | 9.33E-02 | 0.05 | 0.56 | 3.57E-01 |
| *CCL3L1* | 17 | rs544198 | 34572555 | e | G | 0.32 | 1.01 | 9.62E-01 | 0.31 | 1.05 | 8.41E-01 | 0.31 | 0.91 | 7.52E-01 |
| *CCL3L1* | 17 | rs657672 | 34572591 | e | A | 0.04 | 0.66 | 4.08E-01 | 0.04 | 0.54 | 4.01E-01 | 0.04 | 1.15 | 8.13E-01 |
| *CCL3L1* | 17 | rs657723 | 34572629 | e | T | 0.31 | 1.07 | 7.42E-01 | 0.31 | 0.77 | 3.33E-01 | 0.31 | 1.20 | 5.27E-01 |
| *CCL3L1* | 17 | rs593772 | 34575487 | g | A | 0.02 | 1.06 | 9.14E-01 | 0.02 | 0.93 | 9.22E-01 | 0.02 | 1.56 | 5.14E-01 |
| *CCL3L1* | 17 | rs16527 | 34575606 | a | A | 0.30 | 1.16 | 4.72E-01 | 0.30 | 0.83 | 4.83E-01 | 0.30 | 1.24 | 4.23E-01 |
| *CCL3L1* | 17 | rs801259 | 34580691 | i | A | 0.40 | 1.07 | 7.19E-01 | 0.40 | 0.75 | 2.57E-01 | 0.40 | 1.24 | 4.43E-01 |
| *CCL3L1* | 17 | rs17633541 | 34583536 | b | A | 0.03 | 0.63 | 4.09E-01 | 0.03 | 0.62 | 5.43E-01 | 0.03 | 0.57 | 4.83E-01 |
| *CCL3L1* | 17 | rs626657 | 34586489 | d | C | 0.43 | 1.22 | 3.06E-01 | 0.42 | 0.97 | 9.01E-01 | 0.42 | 1.00 | 9.96E-01 |
| *CCL3L1* | 17 | rs16531 | 34603181 | d | G | 0.40 | 0.77 | 1.62E-01 | 0.40 | 0.83 | 4.33E-01 | 0.40 | 0.96 | 9.01E-01 |
| *CCL3L1* | 17 | rs521633 | 34608592 | i | C | 0.03 | 0.52 | 3.43E-01 | 0.02 | 0.43 | 4.30E-01 | 0.02 | 0.63 | 6.83E-01 |
| *CCL3L1* | 17 | rs16530 | 34610560 | d | A | 0.36 | 0.76 | 1.72E-01 | 0.36 | 0.68 | 1.38E-01 | 0.36 | 1.00 | 9.95E-01 |
| *CCL3L1* | 17 | rs16539 | 34618157 | b | C | 0.34 | 0.68 | 5.49E-02 | 0.35 | 0.69 | 1.60E-01 | 0.35 | 0.88 | 6.71E-01 |
| *CCL3L1* | 17 | rs486512 | 34619225 | b | T | 0.03 | 0.79 | 7.14E-01 | 0.02 | 0.98 | 9.77E-01 | 0.02 | 0.50 | 5.24E-01 |
| *CD209* | 19 | rs12611071 | 7706361 | i | G | 0.34 | 1.16 | 4.44E-01 | 0.35 | 1.33 | 2.41E-01 | 0.35 | 0.85 | 5.69E-01 |
| *CD209* | 19 | rs7248637 | 7713027 | a | T | 0.45 | 0.77 | 1.55E-01 | 0.44 | 0.59 | 2.96E-02 | 0.44 | 0.98 | 9.35E-01 |
| *CD209* | 19 | rs17159889 | 7715631 | d | A | 0.19 | 1.03 | 9.02E-01 | 0.19 | 0.61 | 1.62E-01 | 0.19 | 1.09 | 8.08E-01 |
| *CD209* | 19 | rs2287886 | 7718536 | i | A | 0.20 | 0.71 | 1.50E-01 | 0.20 | 0.64 | 1.68E-01 | 0.20 | 1.14 | 6.86E-01 |
| *CD209* | 19 | rs735240 | 7719336 | i | T | 0.30 | 1.02 | 9.33E-01 | 0.30 | 1.06 | 8.29E-01 | 0.30 | 0.94 | 8.43E-01 |
| *CD209* | 19 | rs10409294 | 7721221 | i | T | 0.16 | 0.86 | 5.83E-01 | 0.17 | 1.06 | 8.56E-01 | 0.17 | 0.77 | 5.19E-01 |
| *CD209* | 19 | rs11881682 | 7721376 | i | T | 0.23 | 1.38 | 1.44E-01 | 0.23 | 1.43 | 1.81E-01 | 0.23 | 1.15 | 6.65E-01 |
| *CD209* | 19 | rs4804805 | 7722546 | i | C | 0.02 | 2.04 | 2.80E-01 | 0.02 | 1.87 | 3.80E-01 | 0.02 | 3.09 | 1.81E-01 |
| *KIR3DL1* | 19 | rs10500318 | 60012591 | d | A | 0.03 | 1.18 | 7.60E-01 | 0.03 | 1.06 | 9.27E-01 | 0.03 | 0.75 | 7.26E-01 |
| *KIR3DL1* | 19 | rs631717 | 60014787 | d | C | 0.35 | 1.07 | 7.23E-01 | 0.35 | 1.08 | 7.34E-01 | 0.35 | 0.80 | 4.27E-01 |
| *KIR3DL1* | 19 | rs649216 | 60016447 | h | T | 0.24 | 1.13 | 5.71E-01 | 0.24 | 1.15 | 6.09E-01 | 0.24 | 0.70 | 2.79E-01 |
| *KIR3DL1* | 19 | rs581623 | 60018551 | i | A | 0.36 | 1.02 | 9.15E-01 | 0.35 | 1.05 | 8.42E-01 | 0.35 | 0.76 | 3.12E-01 |
| *KIR3DL1* | 19 | rs1654644 | 60065174 | d | G | 0.49 | 0.69 | 4.27E-02 | 0.49 | 0.65 | 7.06E-02 | 0.49 | 0.82 | 4.63E-01 |
| *KIR3DL1* | 19 | rs3826878 | 60069023 | d | G | 0.02 | 2.40 | 2.30E-01 | 0.02 | 0.50 | 5.32E-01 | 0.02 | 1.66 | 5.44E-01 |
| *KIR3DL1* | 19 | rs3745902 | 60069820 | e | A | 0.15 | 0.85 | 5.18E-01 | 0.15 | 0.74 | 3.81E-01 | 0.15 | 0.88 | 7.06E-01 |
| *KIR3DL1* | 19 | rs17771967 | 60072026 | b | G | 0.45 | 0.77 | 1.67E-01 | 0.45 | 0.61 | 5.13E-02 | 0.45 | 0.89 | 6.88E-01 |
| *KIR3DL1* | 19 | rs11672983 | 60074863 | i | A | 0.31 | 0.70 | 7.81E-02 | 0.30 | 0.65 | 1.03E-01 | 0.30 | 0.82 | 4.74E-01 |
| *KIR3DL1* | 19 | rs11665986 | 60075753 | i | A | 0.08 | 0.73 | 3.62E-01 | 0.08 | 1.31 | 4.83E-01 | 0.08 | 0.38 | 1.46E-01 |
| *SDC4* | 20 | rs736389 | 43380182 | i | C | 0.04 | 0.87 | 7.83E-01 | 0.04 | 1.11 | 8.64E-01 | 0.04 | 0.68 | 6.35E-01 |
| *SDC4* | 20 | rs11698812 | 43381635 | i | C | 0.42 | 0.92 | 6.49E-01 | 0.42 | 1.06 | 8.03E-01 | 0.42 | 0.93 | 7.99E-01 |
| *SDC4* | 20 | rs11696248 | 43384230 | i | G | 0.14 | 1.28 | 3.84E-01 | 0.14 | 1.69 | 1.12E-01 | 0.14 | 1.28 | 5.59E-01 |
| *SDC4* | 20 | rs6073708 | 43386291 | b | A | 0.30 | 0.79 | 2.54E-01 | 0.31 | 0.96 | 8.73E-01 | 0.31 | 0.80 | 4.45E-01 |
| *SDC4* | 20 | rs6104118 | 43386344 | b | T | 0.42 | 1.15 | 4.69E-01 | 0.42 | 1.09 | 7.16E-01 | 0.42 | 1.18 | 5.33E-01 |
| *SDC4* | 20 | rs6073718 | 43398690 | d | G | 0.38 | 0.98 | 9.17E-01 | 0.37 | 0.89 | 6.28E-01 | 0.37 | 0.89 | 6.94E-01 |
| *SDC4* | 20 | rs2284277 | 43400724 | d | A | 0.13 | 1.33 | 3.25E-01 | 0.13 | 1.66 | 1.27E-01 | 0.13 | 0.90 | 8.28E-01 |
| *SDC4* | 20 | rs11905122 | 43402020 | d | C | 0.37 | 1.04 | 8.36E-01 | 0.36 | 0.91 | 7.12E-01 | 0.36 | 0.91 | 7.27E-01 |
| *SDC4* | 20 | rs2267868 | 43403254 | d | G | 0.03 | 0.77 | 6.36E-01 | 0.03 | 0.94 | 9.32E-01 | 0.03 | 1.47 | 5.93E-01 |
| *SDC4* | 20 | rs2267869 | 43403791 | d | A | 0.48 | 1.09 | 6.55E-01 | 0.48 | 1.01 | 9.55E-01 | 0.48 | 1.05 | 8.54E-01 |
| *SDC4* | 20 | rs8115680 | 43406861 | d | A | 0.24 | 0.82 | 3.58E-01 | 0.24 | 0.57 | 6.74E-02 | 0.24 | 0.82 | 4.99E-01 |
| *SDC4* | 20 | rs1981431 | 43408865 | d | A | 0.07 | 0.86 | 7.10E-01 | 0.07 | 0.79 | 6.58E-01 | 0.07 | 0.45 | 2.03E-01 |
| *SDC4* | 20 | rs1981429 | 43409107 | d | A | 0.31 | 0.84 | 3.77E-01 | 0.30 | 0.60 | 6.67E-02 | 0.30 | 0.71 | 2.39E-01 |
| *SDC4* | 20 | rs4458268 | 43410405 | e | G | 0.05 | 1.11 | 8.14E-01 | 0.05 | 0.84 | 7.67E-01 | 0.05 | 0.64 | 4.99E-01 |
| *SDC4* | 20 | rs1008953 | 43414140 | i | A | 0.17 | 0.96 | 8.77E-01 | 0.17 | 0.91 | 7.67E-01 | 0.17 | 1.34 | 3.91E-01 |
| *APOBEC3G* | 22 | rs5750726 | 37759407 | i | C | 0.19 | 0.87 | 5.79E-01 | 0.20 | 0.98 | 9.40E-01 | 0.20 | 1.00 | 9.93E-01 |
| *APOBEC3G* | 22 | rs4821862 | 37765703 | h | G | 0.45 | 1.03 | 9.02E-01 | 0.45 | 0.89 | 6.36E-01 | 0.45 | 1.04 | 8.89E-01 |
| *APOBEC3G* | 22 | rs2014881 | 37769880 | d | T | 0.16 | 0.78 | 3.66E-01 | 0.16 | 1.10 | 7.83E-01 | 0.16 | 0.82 | 6.29E-01 |
| *APOBEC3G* | 22 | rs2076101 | 37770054 | e | A | 0.16 | 0.76 | 3.25E-01 | 0.16 | 1.08 | 8.19E-01 | 0.16 | 0.82 | 6.29E-01 |
| *APOBEC3G* | 22 | rs12160242 | 37798347 | d | C | 0.14 | 1.21 | 5.00E-01 | 0.14 | 1.39 | 3.13E-01 | 0.14 | 1.38 | 3.99E-01 |
| *APOBEC3G* | 22 | rs8177832 | 37802066 | e | G | 0.38 | 0.98 | 9.29E-01 | 0.38 | 0.60 | 6.26E-02 | 0.38 | 0.88 | 6.69E-01 |
| *APOBEC3G* | 22 | rs5995668 | 37809712 | i | T | 0.24 | 1.08 | 7.41E-01 | 0.24 | 1.05 | 8.69E-01 | 0.24 | 1.02 | 9.62E-01 |
| *APOBEC3G* | 22 | rs17000751 | 37815633 | i | G | 0.34 | 0.80 | 2.77E-01 | 0.34 | 1.09 | 7.31E-01 | 0.34 | 0.69 | 2.34E-01 |

† CHR: Chromosome, BP: Base pair location, A1: risk allele, MAF: Minor Allele Frequency, OR: Odds Ratio, *p*: adjusted by maternal HIV viral load *p*-value. Type: a=3 prime UTR; b=downstream; c=intergenic; d=intronic; e=nonsynonymous coding; f=nonsynonymous coding, splice site; g=splice site, intronic; h=synonymous coding; i=upstream; j=within non coding gene.
