# Supplementary material for: Correction: A whole genome association study of mother-to-child transmission of HIV in Malawi
Source: Genome Med. 2010 Oct 11;2(10):76. doi: 10.1186/gm197 (PMC3092107; doi:10.1186/gm197)
Supplement: Additional file 1 — A Word document giving effect estimates for top SNPs of interest, by mode of transmission. The data provided represent the genome-wide association analysis by mode of HIV transmission. [file gm197-S1.DOCX]

Additional file 1. Effect estimates for top SNPs of interest, by mode of transmission

|  |  |  | **Intrauterine Transmission** | | **Intrapartum Transmission** | |
| --- | --- | --- | --- | --- | --- | --- |
| **CHR** | **SNP** | **A1** | **OR (95%CI)** | ***p*** | **OR (95% CI)** | ***p*** |
| 17 | rs12306 | A | 0.66 (0.36, 1.21) | 1.74E-01 | 0.32 (0.14, 0.70) | 4.39E-03 |
| 8 | rs476321 | T | 1.53 (0.95, 2.46) | 8.37E-02 | 2.39 (1.32, 4.30) | 3.82E-03 |
| 6 | rs2268993 | C | 1.31 (0.78, 2.21) | 3.06E-01 | 3.12 (1.62, 6.03) | 6.95E-04 |
| 18 | rs8084223 | T | 0.35 (0.15, 0.83) | 1.66E-02 | 0.27 (0.10, 0.71) | 7.68E-03 |
| 23 | rs5934013 | G | 1.87 (1.00 , 3.53) | 5.15E-02 | 3.45 (1.49, 8.00) | 3.87E-03 |
| 8 | rs9314565 | G | 0.46 (0.28 , 0.77) | 3.12E-03 | 0.38 (0.21, 0.71) | 2.13E-03 |
| 3 | rs4234621 | C | 0.78 (0.46, 1.33) | 3.67E-01 | 0.18 (0.07, 0.44) | 1.55E-04 |
| 14 | rs2287652 | C | 0.52 (0.26, 1.03) | 6.21E-02 | 0.41 (0.19, 0.92) | 3.03E-02 |
| 9 | rs1889055 | C | 1.85 (1.14, 3.01) | 1.35E-02 | 2.63 (1.49, 4.65) | 8.90E-04 |
| 7 | rs216743 | A | 2.33 (1.20, 4.54) | 1.28E-02 | 4.28 (1.75, 10.48) | 1.44E-03 |
| 7 | rs216744 | G | 2.33 (1.20 , 4.54) | 1.28E-02 | 4.28 (1.75, 10.48) | 1.44E-03 |
| 22 | rs131817 | T | 0.46 (0.24, 0.89) | 1.98E-02 | 0.51 (0.25, 1.01) | 5.18E-02 |
| 7 | rs4722999 | C | 1.72 (1.03, 2.88) | 3.73E-02 | 1.79 (0.99, 3.25) | 5.45E-02 |
| 17 | rs8069770 | T | 0.33 (0.14, 0.81) | 1.56E-02 | 0.40 (0.16, 0.99) | 4.66E-02 |
| 5 | rs6884962 | G | 1.86 (1.15, 2.99) | 1.09E-02 | 1.73 (1.02, 2.95) | 4.36E-02 |
| 12 | rs12579934 | T | 2.01 (1.23, 3.31) | 5.61E-03 | 4.79 (2.39, 9.58) | 9.75E-06 |
| 9 | rs12376718 | T | 1.76 (0.98, 3.15) | 5.85E-02 | 3.18 (1.58, 6.42) | 1.22E-03 |
| 16 | rs6540013 | G | 0.66 (0.40, 1.07) | 8.97E-02 | 0.61 (0.35, 1.08) | 8.78E-02 |
| 16 | rs12598821 | T | 0.78 (0.48, 1.26) | 3.05E-01 | 0.34 (0.18, 0.63) | 7.65E-04 |
| 1 | rs3861824 | A | 0.29 (0.10, 0.84) | 2.21E-02 | 0.31 (0.10, 0.99) | 4.85E-02 |

† Effect estimates and *p*-values for intrauterine and intrapartum HIV transmission phenotypes, summarized for the 20 most-significant SNPs from cumulative HIV MTCT analyses. CHR: Chromosome, A1: risk allele designated by *PLINK*, MAF: Minor Allele Frequency, OR: Odds Ratio, 95% CI: 95% Confidence Interval of the OR, *p*: adjusted by maternal HIV viral load *p*-value.
