# Supplementary material for: Correction: A whole genome association study of mother-to-child transmission of HIV in Malawi
Source: Genome Med. 2010 Oct 11;2(10):76. doi: 10.1186/gm197 (PMC3092107; doi:10.1186/gm197)
Supplement: Additional file 2 — A Word document giving effect estimates for SNPs near or within genes associated with HIV/AIDS. The data provided represent the genome-wide association analysis for specific regions that have previously demonstrated association with HIV/AIDS, described in the Introduction section. [file gm197-S2.DOCX]

Additional file 2. Effect estimates for SNPs near or within genes associated with HIV/AIDS

|  |  |  |  |  |  |  | **Cumulative HIV MTCT** | | **Intrauterine Transmission** | | **Intrapartum Transmission** | |
| --- | --- | --- | --- | --- | --- | --- | --- | --- | --- | --- | --- | --- |
| **Within 10KB**  **of Gene:** | **CHR** | **SNP** | **BP** | **Type** | **A1** | **MAF** | **OR** | ***p*** | **OR** | ***p*** | **OR** | ***p*** |
| *SDC3* | 1 | rs6425688 | 31333599 | d | C | 0.10 | 1.43 | 2.32E-01 | 0.83 | 6.41E-01 | 2.15 | 4.66E-02 |
| *SDC3* | 1 | rs2796208 | 31335379 | d | C | 0.08 | 0.96 | 9.06E-01 | 1.11 | 8.05E-01 | 0.81 | 6.90E-01 |
| *SDC3* | 1 | rs2506969 | 31336439 | d | C | 0.10 | 0.88 | 7.00E-01 | 1.13 | 7.47E-01 | 0.82 | 6.88E-01 |
| *SDC3* | 1 | rs12408228 | 31337417 | d | A | 0.29 | 1.06 | 7.61E-01 | 0.99 | 9.71E-01 | 0.88 | 6.56E-01 |
| *SDC3* | 1 | rs4949320 | 31339570 | d | T | 0.33 | 1.07 | 7.32E-01 | 1.32 | 2.53E-01 | 0.81 | 4.73E-01 |
| *SDC3* | 1 | rs3766286 | 31344250 | a | C | 0.37 | 1.10 | 6.39E-01 | 1.12 | 6.53E-01 | 0.76 | 3.74E-01 |
| *SDC3* | 1 | rs2488238 | 31344952 | a | A | 0.15 | 1.02 | 9.38E-01 | 1.26 | 4.97E-01 | 0.67 | 3.67E-01 |
| *SDC3* | 1 | rs3820088 | 31345747 | a | A | 0.32 | 1.09 | 6.71E-01 | 1.24 | 3.85E-01 | 0.71 | 2.89E-01 |
| *SDC3* | 1 | rs2282440 | 31347320 | g | T | 0.02 | 0.78 | 7.35E-01 | 0.58 | 6.13E-01 | 0.60 | 6.50E-01 |
| *SDC3* | 1 | rs4949184 | 31347399 | g | A | 0.34 | 1.10 | 6.24E-01 | 1.11 | 6.89E-01 | 0.75 | 3.69E-01 |
| *SDC3* | 1 | rs2491133 | 31350566 | b | T | 0.08 | 0.41 | 2.88E-02 | 0.66 | 4.03E-01 | 0.35 | 9.62E-02 |
| *SDC3* | 1 | rs1891419 | 31351513 | j | A | 0.05 | 0.84 | 7.17E-01 | 0.90 | 8.66E-01 | 0.57 | 4.84E-01 |
| *SDC3* | 1 | rs6695189 | 31354077 | f | A | 0.03 | 1.99 | 1.76E-01 | 0.92 | 8.90E-01 | 3.73 | 2.66E-02 |
| *SDC3* | 1 | rs16834123 | 31354164 | f | C | 0.26 | 0.89 | 5.90E-01 | 0.71 | 2.40E-01 | 0.90 | 7.37E-01 |
| *SDC3* | 1 | rs10753239 | 31362224 | f | T | 0.08 | 0.53 | 8.98E-02 | 0.71 | 4.64E-01 | 0.59 | 3.06E-01 |
| *SDC3* | 1 | rs7529390 | 31372686 | f | T | 0.12 | 0.78 | 3.96E-01 | 1.26 | 4.99E-01 | 0.84 | 6.93E-01 |
| *SDC3* | 1 | rs12085929 | 31372734 | f | C | 0.43 | 0.96 | 8.40E-01 | 0.82 | 4.16E-01 | 1.26 | 4.02E-01 |
| *SDC3* | 1 | rs11810325 | 31375569 | f | A | 0.04 | 1.39 | 4.73E-01 | 0.75 | 6.52E-01 | 2.54 | 7.65E-02 |
| *SDC3* | 1 | rs10158813 | 31378286 | f | C | 0.13 | 0.69 | 2.16E-01 | 0.63 | 2.50E-01 | 0.68 | 3.77E-01 |
| *SDC3* | 1 | rs6680835 | 31387330 | e | A | 0.21 | 0.91 | 6.90E-01 | 1.51 | 1.38E-01 | 0.46 | 5.83E-02 |
| *SDC3* | 1 | rs12097621 | 31387356 | e | C | 0.15 | 1.09 | 7.41E-01 | 1.16 | 6.43E-01 | 0.96 | 9.04E-01 |
| *SDC3* | 1 | rs6425689 | 31387718 | e | C | 0.05 | 1.83 | 1.51E-01 | 0.99 | 9.88E-01 | 3.46 | 2.00E-02 |
| *SDC3* | 1 | rs10914232 | 31388559 | e | C | 0.44 | 0.81 | 2.76E-01 | 0.71 | 1.58E-01 | 0.90 | 6.79E-01 |
| *SDC1* | 2 | rs2881925 | 20390694 | e | A | 0.25 | 0.98 | 9.21E-01 | 1.22 | 4.79E-01 | 0.83 | 5.83E-01 |
| *SDC1* | 2 | rs2881926 | 20391853 | e | A | 0.30 | 1.03 | 8.99E-01 | 1.37 | 2.30E-01 | 0.88 | 6.82E-01 |
| *SDC1* | 2 | rs6749689 | 20396122 | d | C | 0.41 | 1.04 | 8.54E-01 | 0.90 | 6.65E-01 | 1.26 | 3.92E-01 |
| *SDC1* | 2 | rs2348476 | 20400056 | d | T | 0.08 | 0.71 | 3.66E-01 | 0.79 | 6.34E-01 | 1.06 | 9.13E-01 |
| *SDC1* | 2 | rs4432408 | 20400300 | d | A | 0.34 | 1.05 | 8.02E-01 | 1.02 | 9.48E-01 | 0.92 | 7.79E-01 |
| *SDC1* | 2 | rs3771254 | 20406245 | f | A | 0.11 | 0.89 | 7.10E-01 | 0.88 | 7.56E-01 | 0.69 | 4.68E-01 |
| *SDC1* | 2 | rs2015110 | 20409322 | f | A | 0.06 | 0.91 | 8.09E-01 | 1.20 | 7.01E-01 | 0.91 | 8.70E-01 |
| *SDC1* | 2 | rs3771240 | 20412686 | f | G | 0.43 | 1.35 | 1.48E-01 | 1.34 | 2.50E-01 | 1.12 | 6.94E-01 |
| *SDC1* | 2 | rs1106111 | 20419112 | f | T | 0.24 | 1.59 | 4.21E-02 | 1.59 | 8.28E-02 | 1.39 | 3.04E-01 |
| *CXCR4* | 2 | rs16832731 | 136864703 | e | G | 0.49 | 1.17 | 4.68E-01 | 1.44 | 1.68E-01 | 1.21 | 5.28E-01 |
| *CXCR4* | 2 | rs16832740 | 136867876 | d | C | 0.19 | 0.58 | 4.28E-02 | 0.40 | 1.73E-02 | 1.10 | 8.00E-01 |
| *CXCR4* | 2 | rs4954391 | 136883823 | e | C | 0.45 | 0.73 | 1.28E-01 | 0.67 | 1.19E-01 | 0.72 | 2.34E-01 |
| *CXCR4* | 2 | rs10191360 | 136884679 | e | C | 0.08 | 1.32 | 4.48E-01 | 2.34 | 3.46E-02 | 0.82 | 7.47E-01 |
| *IL8* | 4 | rs4694636 | 74598809 | e | T | 0.13 | 0.92 | 7.90E-01 | 1.03 | 9.42E-01 | 1.34 | 4.61E-01 |
| *IL8* | 4 | rs16849934 | 74600673 | e | C | 0.12 | 0.95 | 8.52E-01 | 0.88 | 7.18E-01 | 0.86 | 7.14E-01 |
| *IL8* | 4 | rs2227538 | 74606375 | b | T | 0.24 | 0.98 | 9.25E-01 | 1.18 | 5.45E-01 | 1.03 | 9.21E-01 |
| *IL8* | 4 | rs1951699 | 74618909 | e | A | 0.18 | 1.08 | 7.43E-01 | 0.65 | 1.86E-01 | 1.06 | 8.75E-01 |
| *IL4* | 5 | rs2243206 | 132001065 | c | T | 0.13 | 0.81 | 4.87E-01 | 1.33 | 4.45E-01 | 0.28 | 2.78E-02 |
| *IL4* | 5 | rs2243220 | 132002761 | c | C | 0.05 | 0.66 | 3.30E-01 | 0.87 | 7.87E-01 | 0.82 | 7.60E-01 |
| *IL4* | 5 | rs2243300 | 132004086 | c | T | 0.02 | 2.05 | 3.33E-01 | 0.54 | 5.74E-01 | 4.53 | 1.07E-01 |
| *IL4* | 5 | rs2243248 | 132008644 | c | C | 0.21 | 0.76 | 2.65E-01 | 0.97 | 9.22E-01 | 0.60 | 1.69E-01 |
| *IL4* | 5 | rs2070874 | 132009710 | b | A | 0.49 | 1.16 | 4.51E-01 | 1.08 | 7.40E-01 | 1.75 | 4.04E-02 |
| *IL4* | 5 | rs2243268 | 132013963 | f | C | 0.34 | 1.02 | 9.05E-01 | 0.99 | 9.67E-01 | 1.46 | 1.88E-01 |
| *IL4* | 5 | rs2243279 | 132016227 | f | T | 0.08 | 0.65 | 2.39E-01 | 0.75 | 5.43E-01 | 0.93 | 8.90E-01 |
| *IL4* | 5 | rs2243288 | 132017944 | f | T | 0.32 | 1.51 | 2.86E-02 | 0.97 | 8.93E-01 | 1.33 | 2.82E-01 |
| *IL4* | 5 | rs2243290 | 132018169 | f | T | 0.40 | 0.92 | 6.41E-01 | 1.00 | 9.87E-01 | 1.22 | 4.51E-01 |
| *HLA-G* | 6 | rs1619379 | 29785235 | c | T | 0.41 | 0.88 | 4.85E-01 | 0.88 | 5.92E-01 | 0.99 | 9.78E-01 |
| *HLA-G* | 6 | rs2743933 | 29786220 | e | G | 0.10 | 0.90 | 7.45E-01 | 0.86 | 7.13E-01 | 0.51 | 2.49E-01 |
| *HLA-G* | 6 | rs2523790 | 29803650 | d | G | 0.49 | 1.14 | 4.68E-01 | 0.96 | 8.49E-01 | 1.36 | 2.40E-01 |
| *HLA-G* | 6 | rs2394180 | 29805199 | e | C | 0.27 | 1.30 | 1.86E-01 | 1.11 | 6.68E-01 | 1.37 | 2.51E-01 |
| *HLA-G* | 6 | rs9258525 | 29806341 | e | C | 0.06 | 0.90 | 8.08E-01 | 0.92 | 8.77E-01 | 1.37 | 5.82E-01 |
| *HLA-G* | 6 | rs2743937 | 29807923 | e | C | 0.49 | 1.18 | 3.72E-01 | 0.98 | 9.28E-01 | 1.41 | 1.88E-01 |
| *HLA-G* | 6 | rs2735007 | 29808199 | e | T | 0.49 | 1.18 | 3.72E-01 | 0.98 | 9.28E-01 | 1.41 | 1.88E-01 |
| *HLA-G* | 6 | rs2735003 | 29808634 | e | C | 0.49 | 1.18 | 3.71E-01 | 0.98 | 9.29E-01 | 1.41 | 1.88E-01 |
| *HLA-A* | 6 | rs2524005 | 29899677 | c | T | 0.10 | 1.03 | 9.21E-01 | 1.08 | 8.35E-01 | 0.50 | 1.67E-01 |
| *HLA-A* | 6 | rs2860580 | 29906691 | c | A | 0.48 | 0.59 | 1.24E-02 | 0.77 | 2.91E-01 | 0.38 | 3.96E-03 |
| *HLA-C* | 6 | rs3130542 | 31232111 | d | T | 0.17 | 1.17 | 5.51E-01 | 1.87 | 3.62E-02 | 0.47 | 1.18E-01 |
| *HLA-C* | 6 | rs2844623 | 31232543 | d | A | 0.16 | 0.76 | 3.11E-01 | 0.65 | 2.24E-01 | 0.99 | 9.80E-01 |
| *HLA-C* | 6 | rs9264508 | 31233214 | d | T | 0.17 | 1.18 | 5.17E-01 | 1.86 | 3.82E-02 | 0.47 | 1.12E-01 |
| *HLA-C* | 6 | rs9264532 | 31234381 | d | G | 0.37 | 0.79 | 2.43E-01 | 1.08 | 7.65E-01 | 0.57 | 7.59E-02 |
| *HLA-C* | 6 | rs2524099 | 31236051 | d | C | 0.32 | 1.05 | 8.17E-01 | 1.22 | 4.32E-01 | 1.02 | 9.41E-01 |
| *HLA-C* | 6 | rs9394047 | 31236250 | d | G | 0.07 | 1.36 | 3.72E-01 | 1.99 | 6.63E-02 | 1.00 | 9.95E-01 |
| *HLA-C* | 6 | rs2074488 | 31240431 | c | A | 0.10 | 0.93 | 8.21E-01 | 0.70 | 4.04E-01 | 1.38 | 4.20E-01 |
| *HLA-C* | 6 | rs2395471 | 31240692 | c | T | 0.45 | 1.24 | 2.86E-01 | 1.13 | 6.30E-01 | 1.39 | 2.65E-01 |
| *HLA-C* | 6 | rs2249742 | 31240721 | c | C | 0.47 | 1.03 | 8.68E-01 | 1.06 | 8.18E-01 | 1.00 | 9.90E-01 |
| *HLA-C* | 6 | rs5010528 | 31241032 | c | G | 0.18 | 1.72 | 3.25E-02 | 1.48 | 1.87E-01 | 1.17 | 6.54E-01 |
| *HLA-C* | 6 | rs13207315 | 31241127 | c | C | 0.12 | 0.86 | 6.14E-01 | 1.04 | 9.16E-01 | 1.20 | 6.39E-01 |
| *HLA-C* | 6 | rs12111032 | 31242191 | c | C | 0.24 | 0.89 | 6.06E-01 | 0.95 | 8.56E-01 | 1.03 | 9.24E-01 |
| *HLA-C* | 6 | rs9461680 | 31243347 | c | A | 0.26 | 0.64 | 4.65E-02 | 0.54 | 4.20E-02 | 0.73 | 3.31E-01 |
| *HLA-C* | 6 | rs2524077 | 31243603 | k | A | 0.16 | 0.76 | 2.82E-01 | 0.78 | 4.43E-01 | 0.66 | 3.08E-01 |
| *HLA-C* | 6 | rs3130696 | 31243884 | k | A | 0.19 | 1.77 | 1.77E-02 | 1.79 | 3.21E-02 | 1.49 | 2.27E-01 |
| *HLA-C* | 6 | rs2524073 | 31244235 | k | A | 0.14 | 0.61 | 8.82E-02 | 0.49 | 8.30E-02 | 0.59 | 2.24E-01 |
| *HLA-C* | 6 | rs2524070 | 31244520 | k | A | 0.22 | 0.65 | 7.69E-02 | 0.60 | 1.09E-01 | 0.81 | 5.17E-01 |
| *HLA-C* | 6 | rs6906846 | 31245736 | k | T | 0.29 | 1.34 | 1.55E-01 | 1.37 | 2.01E-01 | 1.17 | 5.98E-01 |
| *HLA-C* | 6 | rs7382297 | 31247067 | c | A | 0.03 | 0.82 | 7.20E-01 | 1.50 | 5.09E-01 | 0.43 | 4.42E-01 |
| *HLA-C* | 6 | rs4386816 | 31247135 | c | C | 0.23 | 1.49 | 8.78E-02 | 1.33 | 3.16E-01 | 0.94 | 8.52E-01 |
| *HLA-B* | 6 | rs3134792 | 31312326 | e | C | 0.05 | 1.16 | 7.41E-01 | 0.82 | 7.32E-01 | 2.26 | 1.31E-01 |
| *HLA-B* | 6 | rs2156875 | 31317347 | d | G | 0.41 | 0.79 | 2.13E-01 | 0.99 | 9.61E-01 | 0.84 | 5.32E-01 |
| *HLA-B* | 6 | rs2523619 | 31318144 | d | C | 0.27 | 0.85 | 4.41E-01 | 0.83 | 4.95E-01 | 1.02 | 9.42E-01 |
| *HLA-B* | 6 | rs2442719 | 31320538 | c | T | 0.44 | 0.83 | 3.34E-01 | 1.01 | 9.58E-01 | 0.85 | 5.65E-01 |
| *HLA-B* | 6 | rs2596503 | 31320810 | c | A | 0.14 | 0.61 | 8.70E-02 | 0.68 | 2.99E-01 | 0.61 | 2.46E-01 |
| *HLA-B* | 6 | rs2596501 | 31321211 | c | C | 0.39 | 0.71 | 8.92E-02 | 0.86 | 5.61E-01 | 0.86 | 6.00E-01 |
| *HLA-B* | 6 | rs1058026 | 31321685 | a | G | 0.19 | 1.50 | 1.12E-01 | 1.33 | 3.55E-01 | 1.44 | 3.08E-01 |
| *HLA-B* | 6 | rs2523608 | 31322559 | f | G | 0.38 | 0.63 | 2.89E-02 | 0.75 | 2.60E-01 | 0.68 | 1.89E-01 |
| *HLA-B* | 6 | rs2523589 | 31327334 | c | G | 0.35 | 0.66 | 3.52E-02 | 0.56 | 2.89E-02 | 0.81 | 4.63E-01 |
| *HLA-B* | 6 | rs2523554 | 31331829 | d | C | 0.16 | 0.85 | 5.33E-01 | 0.71 | 3.19E-01 | 1.15 | 7.09E-01 |
| *HLA-B* | 6 | rs2596551 | 31332239 | d | C | 0.19 | 1.44 | 1.42E-01 | 1.34 | 3.23E-01 | 1.16 | 6.95E-01 |
| *HLA-B* | 6 | rs2844575 | 31334945 | c | C | 0.49 | 0.90 | 5.65E-01 | 0.86 | 5.12E-01 | 0.93 | 7.77E-01 |
| *SDC2* | 8 | rs10808350 | 97495996 | e | T | 0.13 | 1.30 | 3.57E-01 | 0.62 | 2.46E-01 | 1.61 | 2.07E-01 |
| *SDC2* | 8 | rs2439516 | 97504741 | c | A | 0.13 | 1.14 | 6.69E-01 | 1.29 | 4.76E-01 | 0.94 | 8.79E-01 |
| *SDC2* | 8 | rs2437770 | 97505545 | c | T | 0.07 | 0.59 | 1.84E-01 | 0.41 | 1.56E-01 | 0.62 | 4.03E-01 |
| *SDC2* | 8 | rs1348563 | 97508361 | f | T | 0.40 | 1.09 | 6.69E-01 | 1.10 | 7.05E-01 | 1.39 | 2.47E-01 |
| *SDC2* | 8 | rs895033 | 97508555 | f | A | 0.24 | 0.77 | 2.62E-01 | 1.04 | 8.96E-01 | 0.89 | 7.13E-01 |
| *SDC2* | 8 | rs13270556 | 97510132 | f | C | 0.12 | 0.76 | 3.81E-01 | 0.62 | 2.50E-01 | 1.28 | 5.56E-01 |
| *SDC2* | 8 | rs12056723 | 97515041 | f | C | 0.12 | 1.06 | 8.54E-01 | 1.48 | 2.48E-01 | 0.47 | 1.48E-01 |
| *SDC2* | 8 | rs2582814 | 97516984 | f | C | 0.18 | 1.86 | 1.30E-02 | 1.14 | 6.64E-01 | 2.13 | 2.86E-02 |
| *SDC2* | 8 | rs2439518 | 97517389 | f | C | 0.39 | 1.52 | 3.79E-02 | 1.06 | 8.22E-01 | 1.44 | 1.98E-01 |
| *SDC2* | 8 | rs2008026 | 97518087 | f | G | 0.15 | 0.68 | 1.66E-01 | 0.57 | 1.44E-01 | 1.00 | 9.97E-01 |
| *SDC2* | 8 | rs2439520 | 97518925 | f | G | 0.25 | 1.03 | 8.85E-01 | 1.09 | 7.59E-01 | 0.89 | 7.10E-01 |
| *SDC2* | 8 | rs2589183 | 97522509 | f | T | 0.23 | 0.70 | 1.25E-01 | 0.92 | 7.61E-01 | 0.85 | 6.09E-01 |
| *SDC2* | 8 | rs2253255 | 97522543 | f | G | 0.19 | 1.00 | 9.88E-01 | 1.23 | 4.71E-01 | 0.66 | 2.61E-01 |
| *SDC2* | 8 | rs6985568 | 97522587 | f | A | 0.10 | 0.90 | 7.60E-01 | 0.72 | 4.67E-01 | 1.17 | 7.47E-01 |
| *SDC2* | 8 | rs2575738 | 97530402 | f | A | 0.30 | 0.89 | 5.88E-01 | 1.46 | 1.55E-01 | 0.45 | 2.05E-02 |
| *SDC2* | 8 | rs2582819 | 97531036 | f | G | 0.44 | 1.23 | 2.71E-01 | 0.97 | 9.07E-01 | 1.77 | 3.60E-02 |
| *SDC2* | 8 | rs2575735 | 97534651 | f | C | 0.37 | 0.84 | 4.12E-01 | 0.60 | 6.47E-02 | 0.98 | 9.49E-01 |
| *SDC2* | 8 | rs2582822 | 97534816 | f | C | 0.33 | 0.86 | 4.45E-01 | 1.29 | 2.90E-01 | 0.56 | 6.17E-02 |
| *SDC2* | 8 | rs2439523 | 97535033 | f | T | 0.05 | 1.00 | 9.93E-01 | 1.96 | 1.63E-01 | 0.38 | 3.36E-01 |
| *SDC2* | 8 | rs2439525 | 97537041 | f | C | 0.18 | 0.99 | 9.67E-01 | 1.77 | 4.77E-02 | 0.53 | 1.26E-01 |
| *SDC2* | 8 | rs7003874 | 97537952 | f | T | 0.16 | 0.94 | 8.02E-01 | 0.53 | 1.01E-01 | 1.54 | 2.10E-01 |
| *SDC2* | 8 | rs2575734 | 97538205 | f | C | 0.39 | 1.05 | 7.95E-01 | 0.96 | 8.49E-01 | 0.78 | 3.63E-01 |
| *SDC2* | 8 | rs2589212 | 97540607 | f | G | 0.22 | 1.14 | 5.59E-01 | 0.95 | 8.59E-01 | 1.78 | 7.10E-02 |
| *SDC2* | 8 | rs2589208 | 97541936 | f | T | 0.47 | 0.92 | 6.52E-01 | 0.99 | 9.63E-01 | 0.63 | 8.23E-02 |
| *SDC2* | 8 | rs895034 | 97543390 | f | C | 0.37 | 0.90 | 6.12E-01 | 0.87 | 5.88E-01 | 0.58 | 6.78E-02 |
| *SDC2* | 8 | rs2440681 | 97544581 | f | T | 0.40 | 1.08 | 7.05E-01 | 0.96 | 8.60E-01 | 1.46 | 2.01E-01 |
| *SDC2* | 8 | rs2437774 | 97547130 | f | T | 0.13 | 1.51 | 1.45E-01 | 1.74 | 8.32E-02 | 0.60 | 2.89E-01 |
| *SDC2* | 8 | rs2589203 | 97549700 | f | G | 0.29 | 0.71 | 1.25E-01 | 0.59 | 7.16E-02 | 0.90 | 7.13E-01 |
| *SDC2* | 8 | rs2589198 | 97556565 | f | C | 0.30 | 1.51 | 4.49E-02 | 1.50 | 9.87E-02 | 1.37 | 2.61E-01 |
| *SDC2* | 8 | rs2582840 | 97558840 | f | G | 0.21 | 1.26 | 3.34E-01 | 1.73 | 5.45E-02 | 1.03 | 9.35E-01 |
| *SDC2* | 8 | rs10100191 | 97559162 | f | G | 0.30 | 0.74 | 1.37E-01 | 0.96 | 8.63E-01 | 0.72 | 2.93E-01 |
| *SDC2* | 8 | rs16894717 | 97559665 | f | A | 0.16 | 1.19 | 5.08E-01 | 0.56 | 1.21E-01 | 2.04 | 4.57E-02 |
| *SDC2* | 8 | rs2582842 | 97563644 | f | C | 0.15 | 0.58 | 4.69E-02 | 0.69 | 2.92E-01 | 0.50 | 1.08E-01 |
| *SDC2* | 8 | rs1444572 | 97564882 | f | G | 0.18 | 0.68 | 1.36E-01 | 0.91 | 7.71E-01 | 0.48 | 7.86E-02 |
| *SDC2* | 8 | rs2582843 | 97566797 | f | G | 0.42 | 0.62 | 1.81E-02 | 0.83 | 4.52E-01 | 0.55 | 4.60E-02 |
| *SDC2* | 8 | rs1984456 | 97567106 | f | A | 0.19 | 0.76 | 2.73E-01 | 0.97 | 9.08E-01 | 0.68 | 3.28E-01 |
| *SDC2* | 8 | rs2016529 | 97578113 | f | G | 0.23 | 1.06 | 7.98E-01 | 0.46 | 1.93E-02 | 1.29 | 4.13E-01 |
| *SDC2* | 8 | rs7831863 | 97583489 | f | A | 0.28 | 1.02 | 9.29E-01 | 1.04 | 8.90E-01 | 0.99 | 9.86E-01 |
| *SDC2* | 8 | rs2575710 | 97583976 | f | T | 0.48 | 0.91 | 6.39E-01 | 1.03 | 9.13E-01 | 0.71 | 2.33E-01 |
| *SDC2* | 8 | rs2514781 | 97586152 | f | T | 0.17 | 1.20 | 4.84E-01 | 1.34 | 3.56E-01 | 1.30 | 5.09E-01 |
| *SDC2* | 8 | rs2464474 | 97597672 | f | C | 0.30 | 0.90 | 6.26E-01 | 0.87 | 6.10E-01 | 1.12 | 7.29E-01 |
| *SDC2* | 8 | rs16894821 | 97604112 | f | C | 0.18 | 1.02 | 9.46E-01 | 0.72 | 3.29E-01 | 0.90 | 7.68E-01 |
| *SDC2* | 8 | rs1126681 | 97605800 | j | T | 0.04 | 0.31 | 4.30E-02 | 0.21 | 1.29E-01 | 0.43 | 2.93E-01 |
| *SDC2* | 8 | rs2704256 | 97607422 | f | G | 0.04 | 0.31 | 4.30E-02 | 0.21 | 1.29E-01 | 0.43 | 2.93E-01 |
| *SDC2* | 8 | rs16892175 | 97607862 | f | C | 0.09 | 1.35 | 3.61E-01 | 1.00 | 9.99E-01 | 0.70 | 4.81E-01 |
| *SDC2* | 8 | rs2455049 | 97608105 | f | C | 0.17 | 1.35 | 2.49E-01 | 1.32 | 3.75E-01 | 1.92 | 1.07E-01 |
| *SDC2* | 8 | rs2704271 | 97608394 | f | C | 0.33 | 0.82 | 3.03E-01 | 0.68 | 1.34E-01 | 0.70 | 2.04E-01 |
| *SDC2* | 8 | rs16894859 | 97610088 | f | G | 0.36 | 0.86 | 4.41E-01 | 1.07 | 7.89E-01 | 0.63 | 1.34E-01 |
| *SDC2* | 8 | rs2582801 | 97610893 | f | C | 0.26 | 1.13 | 5.68E-01 | 1.14 | 6.13E-01 | 1.37 | 2.98E-01 |
| *SDC2* | 8 | rs1561158 | 97615235 | f | A | 0.09 | 0.75 | 3.64E-01 | 0.41 | 9.86E-02 | 1.15 | 7.24E-01 |
| *SDC2* | 8 | rs2575741 | 97619004 | f | A | 0.07 | 0.66 | 2.92E-01 | 0.94 | 9.04E-01 | 0.48 | 2.45E-01 |
| *SDC2* | 8 | rs2651458 | 97625450 | d | C | 0.21 | 1.04 | 8.57E-01 | 1.00 | 9.92E-01 | 1.42 | 2.79E-01 |
| *SDC2* | 8 | rs10955078 | 97629906 | e | C | 0.24 | 1.23 | 3.41E-01 | 1.63 | 6.27E-02 | 1.21 | 5.79E-01 |
| *SDC2* | 8 | rs10090346 | 97630529 | e | A | 0.45 | 1.13 | 5.07E-01 | 0.89 | 6.06E-01 | 1.49 | 1.42E-01 |
| *SDC2* | 8 | rs714046 | 97631573 | e | A | 0.25 | 0.68 | 9.40E-02 | 1.09 | 7.53E-01 | 0.39 | 1.47E-02 |
| *CXCL12* | 10 | rs266105 | 44855663 | e | A | 0.21 | 1.49 | 8.22E-02 | 1.55 | 9.69E-02 | 1.42 | 3.15E-01 |
| *CXCL12* | 10 | rs10900029 | 44862220 | d | T | 0.12 | 1.07 | 8.12E-01 | 0.55 | 1.56E-01 | 1.32 | 4.96E-01 |
| *CXCL12* | 10 | rs266094 | 44864300 | d | A | 0.26 | 1.30 | 2.14E-01 | 1.52 | 9.81E-02 | 0.91 | 7.83E-01 |
| *CXCL12* | 10 | rs1065297 | 44865976 | a | G | 0.10 | 1.49 | 2.07E-01 | 1.67 | 1.53E-01 | 1.41 | 4.42E-01 |
| *CXCL12* | 10 | rs197452 | 44870240 | f | T | 0.10 | 1.09 | 7.81E-01 | 1.16 | 6.81E-01 | 1.21 | 6.60E-01 |
| *CXCL12* | 10 | rs266087 | 44871062 | f | A | 0.26 | 0.83 | 4.06E-01 | 0.55 | 5.76E-02 | 1.04 | 9.08E-01 |
| *CXCL12* | 10 | rs2297630 | 44871548 | f | T | 0.01 | 0.32 | 3.16E-01 | 0.00 | 9.99E-01 | 1.18 | 8.89E-01 |
| *CXCL12* | 10 | rs266086 | 44871833 | f | A | 0.10 | 1.58 | 1.53E-01 | 1.84 | 7.93E-02 | 1.31 | 5.51E-01 |
| *CXCL12* | 10 | rs4948878 | 44874821 | f | C | 0.04 | 1.92 | 1.56E-01 | 2.19 | 1.01E-01 | 2.44 | 1.55E-01 |
| *CXCL12* | 10 | rs2839692 | 44874867 | f | G | 0.20 | 0.86 | 5.36E-01 | 1.02 | 9.56E-01 | 0.73 | 3.99E-01 |
| *CXCL12* | 10 | rs2839690 | 44875166 | f | C | 0.20 | 0.86 | 5.36E-01 | 1.02 | 9.56E-01 | 0.73 | 3.99E-01 |
| *CXCL12* | 10 | rs11592974 | 44877501 | f | C | 0.10 | 0.87 | 6.69E-01 | 0.95 | 9.05E-01 | 0.92 | 8.57E-01 |
| *CXCL12* | 10 | rs3780891 | 44878713 | h | A | 0.02 | 0.17 | 9.58E-02 | 0.54 | 5.64E-01 | 0.00 | 9.98E-01 |
| *CXCL12* | 10 | rs2861442 | 44884959 | c | G | 0.20 | 0.67 | 9.86E-02 | 0.86 | 6.16E-01 | 0.63 | 2.09E-01 |
| *CXCL12* | 10 | rs1855531 | 44889652 | e | A | 0.04 | 0.89 | 7.97E-01 | 0.74 | 6.27E-01 | 2.12 | 2.18E-01 |
| *MBL2* | 10 | rs3829168 | 54515590 | c | G | 0.13 | 1.21 | 5.12E-01 | 0.80 | 5.57E-01 | 1.08 | 8.53E-01 |
| *MBL2* | 10 | rs10508975 | 54516331 | c | G | 0.06 | 1.02 | 9.58E-01 | 1.47 | 3.79E-01 | 0.82 | 7.39E-01 |
| *MBL2* | 10 | rs7098284 | 54517672 | c | C | 0.34 | 1.20 | 3.49E-01 | 1.06 | 8.12E-01 | 1.30 | 3.44E-01 |
| *MBL2* | 10 | rs10740519 | 54517687 | c | T | 0.43 | 0.86 | 4.12E-01 | 1.00 | 9.92E-01 | 0.75 | 3.08E-01 |
| *MBL2* | 10 | rs920727 | 54518968 | c | A | 0.31 | 0.97 | 8.67E-01 | 1.24 | 3.65E-01 | 0.79 | 4.00E-01 |
| *MBL2* | 10 | rs2099902 | 54525849 | a | A | 0.37 | 0.82 | 2.84E-01 | 1.08 | 7.38E-01 | 0.78 | 3.52E-01 |
| *MBL2* | 10 | rs2120132 | 54526040 | a | C | 0.46 | 1.26 | 2.06E-01 | 0.98 | 9.13E-01 | 1.25 | 3.96E-01 |
| *MBL2* | 10 | rs10824792 | 54526206 | a | T | 0.10 | 0.94 | 8.52E-01 | 1.09 | 8.31E-01 | 0.70 | 4.67E-01 |
| *MBL2* | 10 | rs10082466 | 54526622 | a | C | 0.47 | 1.35 | 1.01E-01 | 1.09 | 7.03E-01 | 1.31 | 2.94E-01 |
| *MBL2* | 10 | rs1838065 | 54529257 | f | C | 0.06 | 1.09 | 8.13E-01 | 1.15 | 7.69E-01 | 0.83 | 7.31E-01 |
| *MBL2* | 10 | rs4935047 | 54530067 | f | C | 0.23 | 1.02 | 9.42E-01 | 0.95 | 8.59E-01 | 0.80 | 4.95E-01 |
| *MBL2* | 10 | rs10824796 | 54534613 | c | A | 0.26 | 0.85 | 4.31E-01 | 0.89 | 6.55E-01 | 0.91 | 7.55E-01 |
| *MBL2* | 10 | rs930506 | 54536551 | e | A | 0.43 | 1.07 | 7.13E-01 | 0.89 | 6.20E-01 | 0.99 | 9.78E-01 |
| *MBL2* | 10 | rs7899547 | 54536839 | e | T | 0.13 | 1.19 | 5.42E-01 | 0.89 | 7.53E-01 | 1.23 | 5.70E-01 |
| *MBL2* | 10 | rs11003132 | 54537447 | e | A | 0.07 | 1.17 | 6.74E-01 | 1.22 | 6.64E-01 | 0.33 | 1.42E-01 |
| *MBL2* | 10 | rs11003134 | 54538159 | e | T | 0.11 | 0.64 | 1.55E-01 | 0.78 | 5.46E-01 | 0.82 | 6.80E-01 |
| *HS3ST3A1* | 17 | rs16947982 | 13389941 | e | G | 0.07 | 1.29 | 4.63E-01 | 1.31 | 5.13E-01 | 1.16 | 7.72E-01 |
| *HS3ST3A1* | 17 | rs16947988 | 13391722 | e | T | 0.06 | 1.34 | 4.40E-01 | 1.19 | 6.98E-01 | 1.62 | 3.47E-01 |
| *HS3ST3A1* | 17 | rs7209938 | 13393882 | e | G | 0.21 | 0.75 | 1.98E-01 | 1.01 | 9.68E-01 | 0.92 | 7.86E-01 |
| *HS3ST3A1* | 17 | rs7216489 | 13395317 | d | T | 0.16 | 1.13 | 6.36E-01 | 1.50 | 1.81E-01 | 0.77 | 5.27E-01 |
| *HS3ST3A1* | 17 | rs6502266 | 13395720 | d | C | 0.04 | 0.51 | 1.95E-01 | 0.68 | 5.51E-01 | 0.22 | 1.53E-01 |
| *HS3ST3A1* | 17 | rs11078161 | 13396044 | d | G | 0.32 | 1.06 | 7.47E-01 | 1.06 | 7.97E-01 | 0.88 | 6.38E-01 |
| *HS3ST3A1* | 17 | rs9909849 | 13398860 | d | C | 0.13 | 0.65 | 1.30E-01 | 1.05 | 8.94E-01 | 0.38 | 6.35E-02 |
| *HS3ST3A1* | 17 | rs1029682 | 13404176 | f | A | 0.05 | 1.36 | 4.59E-01 | 1.78 | 2.15E-01 | 0.62 | 5.39E-01 |
| *HS3ST3A1* | 17 | rs6502269 | 13404781 | f | G | 0.05 | 1.36 | 4.59E-01 | 1.78 | 2.15E-01 | 0.62 | 5.39E-01 |
| *HS3ST3A1* | 17 | rs1860068 | 13408098 | f | G | 0.01 | 0.96 | 9.62E-01 | 1.10 | 9.30E-01 | 1.78 | 6.44E-01 |
| *HS3ST3A1* | 17 | rs3785710 | 13413111 | f | T | 0.11 | 1.65 | 9.87E-02 | 0.97 | 9.46E-01 | 1.63 | 2.12E-01 |
| *HS3ST3A1* | 17 | rs8073109 | 13425423 | f | C | 0.47 | 1.11 | 5.88E-01 | 1.14 | 5.67E-01 | 0.79 | 3.94E-01 |
| *HS3ST3A1* | 17 | rs9896557 | 13435548 | f | C | 0.05 | 0.36 | 4.49E-02 | 0.00 | 9.97E-01 | 0.88 | 8.33E-01 |
| *HS3ST3A1* | 17 | rs9897001 | 13435732 | f | A | 0.32 | 0.82 | 3.41E-01 | 0.70 | 1.74E-01 | 1.34 | 3.03E-01 |
| *HS3ST3A1* | 17 | rs11651136 | 13435931 | f | G | 0.29 | 0.83 | 3.70E-01 | 0.76 | 3.07E-01 | 1.39 | 2.44E-01 |
| *HS3ST3A1* | 17 | rs12941565 | 13442366 | f | T | 0.22 | 1.08 | 7.10E-01 | 1.23 | 4.31E-01 | 0.77 | 4.17E-01 |
| *HS3ST3A1* | 17 | rs9916623 | 13443798 | f | C | 0.46 | 0.86 | 4.22E-01 | 0.86 | 5.33E-01 | 1.18 | 5.41E-01 |
| *HS3ST3A1* | 17 | rs7224721 | 13444373 | f | G | 0.38 | 1.23 | 2.83E-01 | 1.38 | 1.67E-01 | 0.94 | 8.20E-01 |
| *HS3ST3A1* | 17 | rs16948063 | 13444681 | f | G | 0.09 | 1.13 | 7.22E-01 | 0.62 | 2.99E-01 | 0.49 | 1.79E-01 |
| *HS3ST3A1* | 17 | rs8082242 | 13448306 | f | C | 0.02 | 1.02 | 9.78E-01 | 0.54 | 5.50E-01 | 1.01 | 9.85E-01 |
| *HS3ST3A1* | 17 | rs3785705 | 13449973 | f | C | 0.02 | 1.02 | 9.78E-01 | 0.54 | 5.50E-01 | 1.01 | 9.85E-01 |
| *HS3ST3A1* | 17 | rs10521225 | 13451365 | f | C | 0.21 | 1.17 | 4.71E-01 | 1.35 | 2.60E-01 | 0.78 | 4.58E-01 |
| *HS3ST3A1* | 17 | rs9941385 | 13451812 | f | G | 0.03 | 0.47 | 2.21E-01 | 0.00 | 9.98E-01 | 1.05 | 9.48E-01 |
| *HS3ST3A1* | 17 | rs10521226 | 13451829 | f | A | 0.35 | 0.82 | 2.92E-01 | 0.62 | 6.00E-02 | 1.51 | 1.22E-01 |
| *HS3ST3A1* | 17 | rs1981629 | 13452856 | f | A | 0.47 | 0.68 | 4.23E-02 | 0.53 | 1.01E-02 | 1.52 | 1.23E-01 |
| *HS3ST3A1* | 17 | rs10521227 | 13453960 | f | T | 0.08 | 0.80 | 5.63E-01 | 0.56 | 2.71E-01 | 1.09 | 8.68E-01 |
| *HS3ST3A1* | 17 | rs3785702 | 13455035 | f | G | 0.41 | 0.87 | 4.58E-01 | 0.63 | 5.05E-02 | 1.19 | 5.04E-01 |
| *HS3ST3A1* | 17 | rs9303081 | 13456721 | f | T | 0.32 | 1.03 | 8.89E-01 | 0.58 | 3.85E-02 | 1.31 | 3.24E-01 |
| *HS3ST3A1* | 17 | rs7212673 | 13458246 | f | T | 0.31 | 0.99 | 9.58E-01 | 0.51 | 1.65E-02 | 1.27 | 3.89E-01 |
| *HS3ST3A1* | 17 | rs6502282 | 13458761 | f | T | 0.17 | 1.08 | 7.43E-01 | 0.55 | 8.62E-02 | 2.48 | 6.48E-03 |
| *HS3ST3A1* | 17 | rs3785697 | 13464187 | f | C | 0.49 | 1.27 | 2.10E-01 | 1.14 | 5.65E-01 | 1.06 | 8.37E-01 |
| *HS3ST3A1* | 17 | rs4399565 | 13466894 | f | C | 0.48 | 0.95 | 8.11E-01 | 1.40 | 1.68E-01 | 0.81 | 4.52E-01 |
| *HS3ST3A1* | 17 | rs2098026 | 13470297 | f | A | 0.35 | 1.00 | 9.90E-01 | 1.63 | 4.86E-02 | 0.78 | 3.92E-01 |
| *HS3ST3A1* | 17 | rs4588021 | 13474747 | f | T | 0.10 | 1.75 | 8.67E-02 | 0.98 | 9.57E-01 | 0.99 | 9.76E-01 |
| *HS3ST3A1* | 17 | rs3785692 | 13476403 | f | A | 0.34 | 1.38 | 1.02E-01 | 1.41 | 1.53E-01 | 1.32 | 2.84E-01 |
| *HS3ST3A1* | 17 | rs16948136 | 13476900 | f | A | 0.49 | 1.20 | 3.22E-01 | 1.30 | 2.62E-01 | 1.21 | 4.57E-01 |
| *HS3ST3A1* | 17 | rs8082581 | 13481453 | f | C | 0.30 | 0.90 | 6.14E-01 | 1.31 | 3.06E-01 | 0.75 | 3.46E-01 |
| *HS3ST3A1* | 17 | rs12952904 | 13487098 | f | A | 0.06 | 1.07 | 8.67E-01 | 1.04 | 9.43E-01 | 0.68 | 5.88E-01 |
| *HS3ST3A1* | 17 | rs11078168 | 13489612 | f | A | 0.29 | 0.66 | 5.79E-02 | 0.53 | 3.03E-02 | 0.54 | 6.08E-02 |
| *HS3ST3A1* | 17 | rs3785690 | 13495749 | f | T | 0.15 | 1.03 | 9.13E-01 | 0.47 | 5.43E-02 | 1.21 | 5.85E-01 |
| *HS3ST3A1* | 17 | rs726799 | 13500396 | f | T | 0.42 | 1.19 | 3.65E-01 | 1.11 | 6.57E-01 | 0.91 | 7.01E-01 |
| *HS3ST3A1* | 17 | rs8074072 | 13507869 | c | G | 0.23 | 1.16 | 4.97E-01 | 1.09 | 7.58E-01 | 1.28 | 4.28E-01 |
| *CCL5* | 17 | rs2291299 | 34191406 | f | C | 0.49 | 0.99 | 9.69E-01 | 0.99 | 9.79E-01 | 0.84 | 5.09E-01 |
| *CCL5* | 17 | rs16971600 | 34196878 | k | G | 0.07 | 0.69 | 2.89E-01 | 1.19 | 6.58E-01 | 0.55 | 3.28E-01 |
| *CCL5* | 17 | rs16963927 | 34204807 | f | C | 0.29 | 0.83 | 3.56E-01 | 0.77 | 3.24E-01 | 0.86 | 6.09E-01 |
| *CCL3* | 17 | rs11651881 | 34406906 | c | A | 0.20 | 1.29 | 2.73E-01 | 1.21 | 4.86E-01 | 1.13 | 6.92E-01 |
| *CCL3* | 17 | rs1851503 | 34412202 | k | C | 0.27 | 1.14 | 5.47E-01 | 0.99 | 9.57E-01 | 0.97 | 9.26E-01 |
| *CCL3* | 17 | rs9972960 | 34420079 | c | A | 0.35 | 1.16 | 4.51E-01 | 0.93 | 7.72E-01 | 1.20 | 5.24E-01 |
| *CCL4* | 17 | rs1634508 | 34426245 | c | T | 0.10 | 1.13 | 6.77E-01 | 1.64 | 1.48E-01 | 0.69 | 4.65E-01 |
| *CCL3* | 17 | rs1634508 | 34426245 | c | T | 0.10 | 1.13 | 6.77E-01 | 1.64 | 1.48E-01 | 0.69 | 4.65E-01 |
| *CCL4* | 17 | rs10491121 | 34430336 | c | T | 0.02 | 0.52 | 3.54E-01 | 0.43 | 4.30E-01 | 0.72 | 7.72E-01 |
| *CCL4* | 17 | rs1619600 | 34434675 | c | C | 0.10 | 0.98 | 9.37E-01 | 1.40 | 3.38E-01 | 0.61 | 3.27E-01 |
| *CCL4* | 17 | rs17617372 | 34434699 | c | A | 0.02 | 1.52 | 5.03E-01 | 1.52 | 5.50E-01 | 0.88 | 8.75E-01 |
| *CCL4* | 17 | rs1357365 | 34436532 | c | A | 0.39 | 1.03 | 8.69E-01 | 1.03 | 9.09E-01 | 1.20 | 5.35E-01 |
| *CCL3L1* | 17 | rs3744350 | 37261879 | f | A | 0.25 | 1.00 | 9.87E-01 | 0.68 | 1.87E-01 | 1.27 | 4.36E-01 |
| *CCL3L1* | 17 | rs3744351 | 37261981 | f | C | 0.28 | 1.26 | 2.81E-01 | 1.19 | 5.20E-01 | 1.32 | 3.56E-01 |
| *CCL3L1* | 17 | rs1989955 | 37262374 | f | G | 0.48 | 0.72 | 9.09E-02 | 0.63 | 6.55E-02 | 0.78 | 4.00E-01 |
| *CCL3L1* | 17 | rs16491 | 37267979 | f | T | 0.41 | 0.85 | 4.35E-01 | 0.64 | 8.01E-02 | 1.06 | 8.37E-01 |
| *CCL3L1* | 17 | rs3025160 | 37268478 | f | T | 0.38 | 0.86 | 4.20E-01 | 0.73 | 1.89E-01 | 1.42 | 1.73E-01 |
| *CCL3L1* | 17 | rs11871779 | 37269770 | f | A | 0.07 | 0.49 | 6.39E-02 | 0.55 | 2.66E-01 | 0.36 | 1.01E-01 |
| *CCL3L1* | 17 | rs16233 | 37270896 | f | C | 0.21 | 0.85 | 4.75E-01 | 0.46 | 2.70E-02 | 0.64 | 2.03E-01 |
| *CCL3L1* | 17 | rs16492 | 37271415 | f | C | 0.05 | 1.78 | 2.13E-01 | 1.29 | 6.40E-01 | 1.02 | 9.73E-01 |
| *CCL3L1* | 17 | rs739823 | 37272571 | f | T | 0.14 | 1.22 | 4.66E-01 | 1.31 | 4.12E-01 | 1.11 | 7.98E-01 |
| *CCL3L1* | 17 | rs3025166 | 37272889 | f | C | 0.01 | 0.98 | 9.79E-01 | 0.00 | 9.99E-01 | 0.95 | 9.65E-01 |
| *CCL3L1* | 17 | rs16235 | 37273131 | f | A | 0.20 | 1.33 | 2.52E-01 | 1.15 | 6.44E-01 | 1.22 | 5.61E-01 |
| *CCL3L1* | 17 | rs16238 | 37275050 | f | C | 0.48 | 1.23 | 2.89E-01 | 0.79 | 3.47E-01 | 1.05 | 8.62E-01 |
| *CCL3L1* | 17 | rs557570 | 37283329 | f | C | 0.27 | 1.55 | 4.67E-02 | 1.58 | 8.09E-02 | 1.14 | 6.72E-01 |
| *CCL3L1* | 17 | rs558317 | 37283370 | f | T | 0.11 | 0.74 | 3.32E-01 | 1.23 | 5.66E-01 | 0.82 | 6.52E-01 |
| *CCL3L1* | 17 | rs16495 | 37288131 | g | A | 0.10 | 1.17 | 6.03E-01 | 1.56 | 2.03E-01 | 0.84 | 6.96E-01 |
| *CCL3L1* | 17 | rs16502 | 37298494 | f | C | 0.08 | 0.86 | 6.86E-01 | 0.75 | 5.56E-01 | 1.12 | 8.11E-01 |
| *CCL3L1* | 17 | rs531728 | 37298761 | f | T | 0.05 | 1.07 | 8.86E-01 | 1.35 | 5.50E-01 | 0.93 | 9.18E-01 |
| *CCL3L1* | 17 | rs16505 | 37300377 | f | A | 0.49 | 0.93 | 6.98E-01 | 1.08 | 7.42E-01 | 0.88 | 6.53E-01 |
| *CCL3L1* | 17 | rs12946701 | 37303198 | f | C | 0.36 | 1.24 | 2.59E-01 | 1.38 | 1.66E-01 | 1.04 | 8.89E-01 |
| *CCL3L1* | 17 | rs16522 | 37316988 | g | G | 0.06 | 0.29 | 1.57E-02 | 0.16 | 7.06E-02 | 0.53 | 3.22E-01 |
| *CCL3L1* | 17 | rs544198 | 37319029 | g | G | 0.32 | 1.01 | 9.81E-01 | 1.00 | 9.97E-01 | 0.86 | 6.14E-01 |
| *CCL3L1* | 17 | rs657672 | 37319065 | g | A | 0.04 | 0.55 | 2.25E-01 | 0.46 | 2.91E-01 | 1.08 | 8.98E-01 |
| *CCL3L1* | 17 | rs657723 | 37319103 | g | T | 0.31 | 1.10 | 6.44E-01 | 0.87 | 5.79E-01 | 1.22 | 4.71E-01 |
| *CCL3L1* | 17 | rs593772 | 37321961 | i | A | 0.02 | 1.05 | 9.30E-01 | 0.91 | 8.96E-01 | 1.58 | 5.00E-01 |
| *CCL3L1* | 17 | rs16527 | 37322080 | b | A | 0.30 | 1.18 | 3.98E-01 | 0.93 | 7.63E-01 | 1.27 | 3.77E-01 |
| *CCL3L1* | 17 | rs801259 | 37327165 | c | A | 0.40 | 1.09 | 6.47E-01 | 0.83 | 4.34E-01 | 1.27 | 3.82E-01 |
| *CCL3L1* | 17 | rs17633541 | 37330010 | d | A | 0.03 | 0.62 | 3.95E-01 | 0.60 | 5.18E-01 | 0.58 | 4.99E-01 |
| *CCL3L1* | 17 | rs626657 | 37332963 | f | C | 0.43 | 1.27 | 2.25E-01 | 1.04 | 8.83E-01 | 1.01 | 9.71E-01 |
| *CCL3L1* | 17 | rs16531 | 37349655 | f | G | 0.40 | 0.79 | 1.96E-01 | 0.87 | 5.59E-01 | 1.00 | 9.92E-01 |
| *CCL3L1* | 17 | rs521633 | 37355066 | c | C | 0.03 | 0.69 | 5.54E-01 | 0.86 | 8.50E-01 | 0.65 | 6.97E-01 |
| *CCL3L1* | 17 | rs16530 | 37357034 | f | A | 0.36 | 0.76 | 1.76E-01 | 0.69 | 1.51E-01 | 1.04 | 9.07E-01 |
| *CCL3L1* | 17 | rs16539 | 37364631 | d | C | 0.34 | 0.67 | 4.61E-02 | 0.67 | 1.23E-01 | 0.91 | 7.50E-01 |
| *CCL3L1* | 17 | rs486512 | 37365699 | d | T | 0.03 | 0.98 | 9.79E-01 | 1.46 | 5.83E-01 | 0.51 | 5.36E-01 |
| *CD209 (DC-SIGN)* | 19 | rs12611071 | 7800361 | c | G | 0.34 | 1.09 | 6.43E-01 | 1.23 | 3.83E-01 | 0.87 | 6.35E-01 |
| *CD209 (DC-SIGN)* | 19 | rs7248637 | 7807027 | a | T | 0.45 | 0.82 | 2.67E-01 | 0.63 | 5.28E-02 | 0.98 | 9.22E-01 |
| *CD209 (DC-SIGN)* | 19 | rs17159889 | 7809631 | f | A | 0.19 | 1.09 | 7.31E-01 | 0.68 | 2.59E-01 | 1.08 | 8.25E-01 |
| *CD209 (DC-SIGN)* | 19 | rs2287886 | 7812536 | c | A | 0.20 | 0.72 | 1.70E-01 | 0.61 | 1.27E-01 | 1.13 | 7.04E-01 |
| *CD209 (DC-SIGN)* | 19 | rs735240 | 7813336 | c | T | 0.30 | 1.00 | 9.98E-01 | 1.06 | 8.32E-01 | 0.97 | 9.17E-01 |
| *CD209 (DC-SIGN)* | 19 | rs10409294 | 7815221 | c | T | 0.16 | 0.85 | 5.41E-01 | 1.02 | 9.46E-01 | 0.79 | 5.60E-01 |
| *CD209 (DC-SIGN)* | 19 | rs11881682 | 7815376 | c | T | 0.23 | 1.43 | 1.07E-01 | 1.53 | 1.04E-01 | 1.18 | 6.12E-01 |
| *CD209 (DC-SIGN)* | 19 | rs4804805 | 7816546 | c | C | 0.02 | 2.01 | 2.90E-01 | 1.82 | 4.03E-01 | 3.15 | 1.74E-01 |
| *KIR3DL1* | 19 | rs10500318 | 55320779 | f | A | 0.03 | 1.33 | 5.88E-01 | 1.40 | 5.79E-01 | 0.76 | 7.44E-01 |
| *KIR3DL1* | 19 | rs631717 | 55322975 | f | C | 0.35 | 1.07 | 7.04E-01 | 1.10 | 6.90E-01 | 0.79 | 4.02E-01 |
| *KIR3DL1* | 19 | rs649216 | 55324635 | j | T | 0.24 | 1.14 | 5.33E-01 | 1.18 | 5.30E-01 | 0.68 | 2.47E-01 |
| *KIR3DL1* | 19 | rs581623 | 55326739 | c | A | 0.36 | 1.03 | 8.94E-01 | 1.06 | 7.95E-01 | 0.75 | 2.93E-01 |
| *KIR3DL1* | 19 | rs1654644 | 55373362 | f | G | 0.49 | 0.68 | 3.67E-02 | 0.65 | 6.81E-02 | 0.81 | 4.30E-01 |
| *KIR3DL1* | 19 | rs3826878 | 55377211 | f | G | 0.02 | 2.37 | 2.37E-01 | 0.49 | 5.16E-01 | 1.69 | 5.30E-01 |
| *KIR3DL1* | 19 | rs3745902 | 55378008 | g | A | 0.15 | 0.84 | 5.05E-01 | 0.77 | 4.41E-01 | 0.87 | 6.89E-01 |
| *KIR3DL1* | 19 | rs17771967 | 55380214 | d | G | 0.45 | 0.76 | 1.50E-01 | 0.62 | 5.21E-02 | 0.88 | 6.41E-01 |
| *KIR3DL1* | 19 | rs11672983 | 55383051 | c | A | 0.31 | 0.71 | 7.75E-02 | 0.67 | 1.19E-01 | 0.80 | 4.16E-01 |
| *KIR3DL1* | 19 | rs11665986 | 55383941 | c | A | 0.08 | 0.78 | 4.52E-01 | 1.43 | 3.42E-01 | 0.39 | 1.54E-01 |
| *SDC4* | 20 | rs736389 | 43946768 | c | C | 0.04 | 0.98 | 9.69E-01 | 1.40 | 5.53E-01 | 0.70 | 6.53E-01 |
| *SDC4* | 20 | rs11698812 | 43948221 | c | C | 0.42 | 0.94 | 7.46E-01 | 1.06 | 8.12E-01 | 0.91 | 7.27E-01 |
| *SDC4* | 20 | rs11696248 | 43950816 | c | G | 0.14 | 1.25 | 4.18E-01 | 1.62 | 1.40E-01 | 1.31 | 5.24E-01 |
| *SDC4* | 20 | rs6073708 | 43952877 | d | A | 0.30 | 0.80 | 2.70E-01 | 0.97 | 9.15E-01 | 0.82 | 4.98E-01 |
| *SDC4* | 20 | rs6104118 | 43952930 | d | T | 0.42 | 1.15 | 4.59E-01 | 1.13 | 6.00E-01 | 1.18 | 5.45E-01 |
| *SDC4* | 20 | rs6073718 | 43965276 | f | G | 0.38 | 0.94 | 7.31E-01 | 0.84 | 4.83E-01 | 0.87 | 6.27E-01 |
| *SDC4* | 20 | rs2284277 | 43967310 | f | A | 0.13 | 1.35 | 2.86E-01 | 1.73 | 9.31E-02 | 0.92 | 8.65E-01 |
| *SDC4* | 20 | rs11905122 | 43968606 | f | C | 0.37 | 0.99 | 9.72E-01 | 0.82 | 4.19E-01 | 0.87 | 6.13E-01 |
| *SDC4* | 20 | rs2267868 | 43969840 | f | G | 0.03 | 0.91 | 8.57E-01 | 1.27 | 6.93E-01 | 1.51 | 5.73E-01 |
| *SDC4* | 20 | rs2267869 | 43970377 | f | A | 0.48 | 1.09 | 6.37E-01 | 0.99 | 9.79E-01 | 1.01 | 9.62E-01 |
| *SDC4* | 20 | rs8115680 | 43973447 | f | A | 0.24 | 0.83 | 3.94E-01 | 0.58 | 6.80E-02 | 0.82 | 5.00E-01 |
| *SDC4* | 20 | rs1981431 | 43975451 | f | A | 0.07 | 0.92 | 8.39E-01 | 0.95 | 9.10E-01 | 0.46 | 2.16E-01 |
| *SDC4* | 20 | rs1981429 | 43975693 | f | A | 0.31 | 0.86 | 4.61E-01 | 0.64 | 9.43E-02 | 0.72 | 2.46E-01 |
| *SDC4* | 20 | rs4458268 | 43976991 | g | G | 0.05 | 1.20 | 6.71E-01 | 1.05 | 9.31E-01 | 0.66 | 5.19E-01 |
| *SDC4* | 20 | rs1008953 | 43980726 | c | A | 0.17 | 1.01 | 9.58E-01 | 1.03 | 9.23E-01 | 1.37 | 3.57E-01 |
| *APOBEC3G (CEM15)* | 22 | rs5750726 | 39434907 | c | C | 0.19 | 0.84 | 4.71E-01 | 0.93 | 8.08E-01 | 0.99 | 9.83E-01 |
| *APOBEC3G (CEM15)* | 22 | rs4821862 | 39441203 | j | G | 0.45 | 1.03 | 8.97E-01 | 0.84 | 4.89E-01 | 1.02 | 9.55E-01 |
| *APOBEC3G (CEM15)* | 22 | rs2014881 | 39445380 | f | T | 0.16 | 0.77 | 3.37E-01 | 1.06 | 8.66E-01 | 0.84 | 6.74E-01 |
| *APOBEC3G (CEM15)* | 22 | rs2076101 | 39445554 | g | A | 0.16 | 0.75 | 2.98E-01 | 1.04 | 9.03E-01 | 0.84 | 6.74E-01 |
| *APOBEC3G (CEM15)* | 22 | rs12160242 | 39473847 | f | C | 0.14 | 1.23 | 4.45E-01 | 1.31 | 4.06E-01 | 1.36 | 4.30E-01 |
| *APOBEC3G (CEM15)* | 22 | rs8177832 | 39477566 | g | G | 0.38 | 1.01 | 9.54E-01 | 0.68 | 1.47E-01 | 0.89 | 7.06E-01 |
| *APOBEC3G (CEM15)* | 22 | rs5995668 | 39485212 | c | T | 0.24 | 1.12 | 6.22E-01 | 1.06 | 8.47E-01 | 1.01 | 9.67E-01 |
| *APOBEC3G (CEM15)* | 22 | rs17000751 | 39491133 | c | G | 0.34 | 0.76 | 1.77E-01 | 1.05 | 8.36E-01 | 0.70 | 2.46E-01 |

† CHR: Chromosome, BP: Base pair location, A1: risk allele, MAF: Minor Allele Frequency, OR: Odds Ratio, *p*: adjusted by maternal HIV viral load *p*-value. Type: a=3 prime UTR; b=3 prime UTR; c=upstream, d=downstream; e=intergenic; f=intronic; g=nonsynonymous coding; h=nonsynonymous coding, splice site; i=splice site, intronic; j=synonymous coding; k=within non coding gene.
